# Optimizing low-carbon strategies in dual-channel supply chains: A quantum game perspective

**DOI:** 10.1371/journal.pone.0323564

**Published:** 2025-06-02

**Authors:** Qi Yao, Yanhui Li, Huan Gao

**Affiliations:** 1 Management School, Wuhan College, Wuhan, Asia, China; 2 School of Information Management, Central China Normal University, Wuhan, Asia, China; Chinese Academy of Sciences Academy of Mathematics and Systems Science, CHINA

## Abstract

The carbon emission management and resource allocation optimization of dual-channel supply chain are of great significance for promoting low-carbon economy and improving supply chain efficiency. In contrast to conventional game theory, Quantum game theory can describe the cooperation and competition of online and offline channels more flexibly through quantum entanglement, so as to overcome the limitations of traditional game theory. We study the application of quantum game theory in low-carbon economy and pricing strategies, and explore how to optimize pricing strategies and improve the low-carbon levels under different entanglement. Therefore, considering consumers’ preference for low-carbon products, this paper establishes a quantum game model to encourage low-carbon actions in the dual-channel supply chain. We discuss three models of centralized decision making, decentralized decision and quantum equilibrium, and analyze the optimal strategies of manufacturers under different entanglement. The results show that quantum game theory has significant advantages in low-carbon dual-channel supply chains. With the increase of quantum entanglement, the low-carbon production level and corporate profitability in the supply chain have been significantly improved, especially under the decentralized decision-making framework, quantum game equilibrium can promote low-carbon behavior and improve profits more than traditional game models. When the degree of entanglement approaches infinity, the total profit of supply chain enterprises using quantum strategy exceeds the profit of supply chain enterprises with centralized decision-making. It is important to note that quantum entanglement can significantly reduce the optimal wholesale and online sales prices for manufacturers. At the same time, the impact of quantum game on manufacturers’ decision-making is more significant than that on retailers, indicating that quantum entanglement shows different sensitivities in influencing the pricing and profitability strategies of different supply chain participants. This finding provides a new theoretical perspective and practical guidance for pricing strategies, firm cooperation and competition in low-carbon supply chains.

## 1. Introduction

As the global climate change problem becomes more serious, low-carbon development has become an important issue for governments and businesses [1]. As a key link in resource consumption and carbon emissions, the low-carbon transformation of supply chains is particularly critical. Governments have formulated and implemented corresponding low-carbon policies and laws and regulations, such as carbon taxes, carbon subsidies and carbon cap-and-trade, to reduce the impact of carbon emissions on the environment [2,3]^.^ Among them, promoting low-carbon production and improving the standardization of low-carbon products are important links to achieve these policy goals [4,5]. To this end, the ecology and environment sector proposes to further improve the standardization of low-carbon products and promote low-carbon consumption, with governments making great efforts to reduce emissions and encourage consumers to purchase low-carbon products through carbon control policies and the promotion of carbon and energy consumption labels.

At the same time, with the growing popularity of Internet applications, supply chain operations are increasingly moving towards dual-channel, i.e., traditional offline channels and emerging online channels. The dual-channel supply chain provides a new way for companies to broaden their sales channels and improve their market competitiveness, but it often comes with additional environmental costs while realizing economic benefits [6]. How to effectively integrate carbon reduction strategies into dual-channel supply chains to achieve a balance between economic and environmental benefits has become an important issue.

Driven by multiple factors, such as policy pressure and consumer preferences, many companies are actively exploring low-carbon transformation to meet the challenges and opportunities driven by both market and policy. For example, major manufacturers such as Haier and Midea have invested heavily in low-carbon production [7]; Ningde Times has established a model for product carbon footprint composition and taken the lead in the industry in conducting full lifecycle carbon footprinting; and a number of global mainstream automotive companies, such as BMW, Mercedes-Benz, Volvo, Audi, Renault and General Motors, have proposed requirements for battery manufacturers on the carbon emissions of battery production. In this context, reducing the carbon footprint of products is not only one of the important measures for companies to achieve sustainable development, but also a key factor to improve market competitiveness [8].

Nevertheless, low-carbon dual-channel supply chains are confronted with a multitude of challenges. The carbon emission characteristics, demand patterns and cost structures of different channels vary considerably, while changes in consumer preferences render the overall supply chain management more challenging. As consumers become more environmentally conscious, they tend to alter their purchasing behaviours in accordance with the carbon performance of each channel [9]. This directly impacts the costs incurred by companies, the low-carbon level of their products and their business decisions [1,9,10]. An understanding of consumer preferences for low-carbon products and different channels enables companies to develop dual-channel carbon reduction strategies that are both accurate and effective. Furthermore, it allows each link in the supply chain to adapt more effectively to the green consumption trend, thereby enhancing the overall low-carbon efficiency of the supply chain.

In an uncertain environment, individual decisions are often influenced by incomplete information, changes in external factors, and the unpredictability of future outcomes. Decisions are further complicated by the fact that individuals make choices in addition to their own preferences and goals, taking into account the reactions of other participants and possible market fluctuations. However, the classical game model usually relies on the deterministic assumption and ignores the uncertainty and complexity in the decision-making process. For example, in complex situations such as epidemic transmission or vaccine supply chain optimization, individual behavior and decision-making are affected by multiple factors, and traditional methods are difficult to fully capture these dynamics [11]. Quantum game theory provides a more flexible framework that can reveal the complexity of individual decisions under uncertain conditions and optimize public decisions, such as collective behavior in COVID-19 prevention and control [12], which helps analyze decision-making behavior in complex environments and optimize cooperation and competition strategies in supply chains.

In order to highlight the uncertainty of complex interaction between enterprises, this paper adopts the unique perspective of quantum game to deeply explore the competition and cooperation relationship between two enterprises, aiming to reveal the application potential of quantum properties in the business field, and provide enterprises with more in-depth and adaptive strategic solutions through this method, so as to better adapt to the competitive and rapidly changing business environment. The introduction of quantum game analysis to the study of low-carbon dual-channel supply chains offers a novel approach to addressing the prevailing issues of management complexity and insufficient synergy mechanisms. The quantum game can more comprehensively portray the interdependence and game relationship between the parties in the dual-channel in terms of carbon emission reduction strategies through the use of quantum characteristics such as superposition and entanglement. In comparison to traditional game models, quantum games are better suited to describe and capture the dynamic synergy and competition between online and offline channels, as well as to reveal the equilibrium strategies of multiple parties in carbon emission reduction and cost optimization [13].

This paper applies quantum game theory to the optimization decision of low-carbon dual-channel supply chain, and innovatively introduces quantum entanglement mechanism to solve the decision conflict and preference coordination problems of all parties in the traditional game model. The model provides a new optimization perspective through the quantum entanglement mechanism, enabling a more efficient balance between low-carbon goals and economic benefits, which has not been fully explored in the existing literature. Quantum game can not only effectively solve the conflict and uncertainty problems in the traditional game methods, but also provide better theoretical support and practical guidance for the decision-making of low-carbon supply chain.

Specifically, (1) Quantum game can overcome the problem of strategic choice conflict in the traditional game, and through the synergistic effect of quantum strategy, all parties can explore new ways of cooperation in the game process, rather than just competitive choice. (2) Quantum games can avoid the limitation of strategy selection in classical game methods by optimizing the decision space of game participants, and its unique non-classical strategy space provides more adaptive decision schemes. (3) The use of quantum entanglement in the quantum game can better capture the strong interdependence between the decisions of the parties in the supply chain decision-making, so that the parties can cooperate in the case of incomplete or uncertain information, and reduce the negative impact of decision conflict. The managerial insights of this paper are derived from the following research questions:

(1) What are the consequences of analyzing the supply chain and its node firms’ low-carbon product production decisions from the perspective of a quantum game? How does this differ from the classical game?
(2) As entanglement increases, is the determination of profit-maximizing optimal pricing for supply chains and their node firms more complex than that of a classical game-theoretic decision?

The remainder of this study is structured as follows: Section 2 provides an overview of the relevant literature, Section 3 presents our model, Section 4 conducts an analysis of the model and performs numerical simulations, and Section 5 offers conclusions and recommendations for future research.

## 2. Literature review

Our research is situated at the confluence of three bodies of literature, including both supply chain management (SCM) and physics. Specifically, it pertains to the domains of dual-channel supply chain pricing and coordination, the reduction of carbon footprint in supply chains, and the application of quantum game theory. Each of these domains is reviewed independently, and our paper is positioned in this paper accordingly.

### 2.1. Dual channel supply chain pricing and coordination

The pricing and coordination of dual-channel supply chains represent crucial focal points in supply chain management, including complicated pricing strategies and coordination mechanisms between manufacturers and retailers. The overarching objective of this study is to maximize the total profit of the entire supply chain while concurrently fostering low-carbon initiatives to attain Pareto optimality.

With respect to pricing in dual-channel supply chains, scholarly attention has been directed towards pricing intricacies in the sales channel to ensure the coordination of dual-channel supply chains. For instance, Li and Mizuno analyzed and compared the structural properties of optimal policies under three different power structures in a dual-channel supply chain, namely Manufacturer Stackelberg, Retailer Stackelberg, and Vertical Nash [14]. The study found that manufacturers and retailers prefer Vertical Nash when wholesale prices are low and Manufacturer Stackelberg when wholesale prices are high. Tran et al. validated the analytical complexity involved in the joint concave of the retailer’s expected profit function with respect to selling price by considering the underlying demand for customer channel choice behavior and attraction between online and physical stores [15]. Datta et al. investigate how to provide the best experience for consumers in dual channels, proposing a dynamic system with dynamic sales efforts and price-sensitive random demand [16]. These studies reveal the complexity and necessity of different power structures, customer channel selection behavior and firm pricing on supply chain management and optimal strategy formulation in dual-channel supply chain.

To realize the coordination of a dual-channel supply chain, scholars have propounded an array of coordination mechanisms, including but not limited to contract coordination and incentive coordination. Of these, the former has garnered the lion’s share of scholarly attention [17,18,19]. The study of these mechanisms helps to motivate manufacturers to expand production capacity, thereby improving the overall performance of the supply chain. Instead, the incentive coordination of cooperation between the two parties is promoted through a reward and punishment system, with the overall goal of maximizing the collective benefits of the supply chain. For example, Song and Zhao studied the coordination of e-commerce supply chains between online sellers and third-party shippers under risk-penalty and flat-rate contracts, aiming to address the challenges of unpredictable demand surges in e-commerce [20]. These results represent a new form of dual marginalization and risk-sharing, in stark contrast to the well-known literature on classic supplier-retailer supply chains. Similarly, Zhang and Xu studied that when suppliers avoid risks, the platform supply chain can realize coordination by implementing incentive subsidies under agency contracts, so as to share risk costs with suppliers and encourage them to improve product quality [21].

In summary, the complicated dynamics of dual-channel supply chain pricing and coordination entail the involvement of manufacturers, retailers, and the entire supply chain. The optimal functioning of a dual-channel supply chain hinges upon the reasonable design of pricing strategies and coordination mechanisms. Although the existing literature has deeply discussed the pricing and coordination mechanism, most studies mainly focus on the traditional economic benefit maximization. It ignores the synergistic optimization of low-carbon goals and environmental benefits and the impact of consumer preferences on economic benefits. Based on consumer product preferences, this paper explores how to achieve a balance between economic and environmental benefits in a low-carbon dual-channel supply chain, especially in the context of carbon emission control and resource allocation.

### 2.2. Reducing the carbon footprint of the supply chain

The decision-making processes related to emission reduction in enterprises have been the subject of extensive literature [22,23,24,25]. For instance, Gao and Souza delved into the options available to enterprises for mitigating their carbon footprint, specifically examining the reduction of controllable carbon emissions and the procurement of carbon offsets, and analyzed their ramifications on corporate decision-making and societal well-being [9]. Zhu et al. examine whether the costs of blockchain operations and construction aimed at increasing the level of corporate carbon reduction are at the expense of squeezing resources previously devoted to carbon reduction (CER) research and development (R&D) or at the expense of corporate profits, highlighting the importance of combining blockchain integration with company-specific characteristics and market conditions to achieve optimized environmental goals [26]. Ding et al. deliberated upon the production, emission reduction, and recycling decisions made by manufacturers operating in both monopolistic and competitive environments, taking into account the implications of carbon taxation and recycling policies. In addition, scholars have assessed the influence of low-carbon policies and corporate decision-making on supply chain coordination under governmental oversight [27]. For instance, Yu et al. explored the effects of carbon tax policies on two competitive supply chains engaged in emission reduction efforts, and formulated a supply chain coordination contract tailored to situations where the collaboration of manufacturers in the two supply chains could potentially undermine the interests of retailers [28].

The implementation of carbon control and carbon offsetting is crucial for advancing emission reduction initiatives. Collaborative efforts are essential for realizing such reduction. For instance, Wang et al. studied the emission reduction decisions of enterprises under non-cooperative, one-way cooperation, and two-way cooperation scenarios [29]. They found that cooperative decision-making improved carbon emission reduction levels and social welfare compared to non-cooperative decision making. Li et al. (2024) considered the impact of carbon tax and tariff on transnational closed-loop supply chains and found that joint emission reduction can maximize supply chain profits [30]. Liu et al. found that cooperative emission reduction between manufacturers and retailers under centralized decision-making can achieve environmental protection and improved profits simultaneously [31].

In summary, extant literature has probed a diverse array of avenues and strategies through which corporate entities may endeavor to curtail their carbon emissions, including facets such as carbon emission reduction, carbon offsets, and the multifaceted impacts of carbon policies on supply chains and societal well-being. This body of work highlights the paramount significance of cooperative emission reduction endeavors and offers a robust theoretical underpinning to guide enterprises in the formulation and execution of sustainable development strategies. Although existing studies have deeply analyzed the impact of low-carbon policies, carbon emission reduction decisions and government regulation on the supply chain, most studies still focus on the traditional game theory framework and lack in-depth discussion on the coordination mechanism of low-carbon supply chain. Especially in the context of low-carbon dual-channel supply chain, how to optimize the strategy of carbon emission reduction and resource allocation through quantum game is still an understudied field. This paper fills this gap and discusses how to optimize carbon emission control and resource allocation in low-carbon dual-channel supply chain by using quantum game theory, so as to promote enterprises to reduce carbon footprint while improving the overall efficiency of supply chain.

### 2.3. Application of quantum game

In recent years, the emerging field of quantum game theory as an autonomous research direction has gained more and more attention. In addition to its wide application in physics, quantum games have been applied in several other fields, such as service supply chain, dual-channel supply chain, investment and innovation, and have obtained equilibrium strategies that are superior to classical strategies [32,33,34,35,36,37,38]. Piotrowski & Sładkowski undertook an exploration of quantum games through the lens of decision theory, drawing upon instances from biology, economics, and gambling, among others, thereby highlighting the relevance of research in these domains to quantum algorithms and protocols [39]. Studies have exhibited that quantum entanglement stands as the pivotal determinant influencing the final measurement result, constituting an efficacious resource in the domain of quantum gaming [40]. The quantum framework adeptly captures the complicated interdependencies among the decision states of all involved parties by virtue of entanglement’s defining attributes.

Classical games provide actions and strategies with the best results, while quantum games study the linear superposition of these strategies. The superposition and entanglement of quantum games expand the strategic space of decision-making participants, making it easier for participants to find the optimal strategy through the selection of quantum strategies. For example, He et al. studied ways to mitigate the betrayal between developers and contractors in the process of green building construction, and used the quantum game method to maximize the interests of both parties and ensure their commitment to green building development [36]. Peng et al. established a game model between financial institutions and energy enterprises, aiming to enhance the green investment intensity of energy enterprises under the background of “One Belt and One Road” energy projects [37]. Meanwhile, Li et al. employed quantum game theory to study the reduction of food loss and waste in collaborative efforts between suppliers and retailers, demonstrating that the level of cooperation between these entities in waste reduction can be enhanced in the maximum entanglement quantum scenarios [41].

In summary, a new quantum game decision model has been explored by certain scholars, drawing upon the research concepts and methodologies of quantum theory. This model aims to explicate issues that are resistant to explanation in the framework of traditional decision theories, particularly individual decision-making behavior in uncertain environments. Although the existing literature shows the application of quantum game in various supply chains, systematic research on quantum game in low-carbon dual-channel supply chains is lacking. This paper aims to explore how quantum games can optimize pricing strategies, resource allocation and carbon emission control of low-carbon dual-channel supply chains through mechanisms such as quantum entanglement, in order to promote the realization of low-carbon economy and improve the overall efficiency of supply chains.

## 3. Problem description and equilibrium solution

This paper postulates the existence of a manufacturer and a retailer in a low-carbon supply chain system. The manufacturer is responsible for producing a singular low-carbon product. On one hand, the manufacturer vends low-carbon products to retailers through conventional retail channels at a wholesale price of w , while retailers sell these products to consumers at a price $p_{r}$. On the other hand, the manufacturer sells the product directly to consumers through online channels at a price $p_{m}$. Although there are many new opinion updating protocols [42,43], we still use the traditional game rules for decision-making process. First, the manufacturer determines the wholesale price and the low carbon level of the product, and then sells the product to retailers. Then, the two companies jointly determine the price of the product in different distribution channels. Consumers generate demand based on price and low carbon level, which ultimately determines the profits of both enterprises.

This paper undertakes an analysis of the manufacturer’s decisions regarding the level of carbon emission and pricing across different supply chain strategies. Under the centralized decision model, the manufacturer and retailer collaborate to maximize the profits of the supply chain. Conversely, under the decentralized decision model, the manufacturer, aiming to maximize its own profits, first determines the wholesale price to retailers and the online sales price, and subsequently establishes the level of carbon emission for the product. Similarly, based on the manufacturer’s decision, the retailer determines the offline sales price to maximize its own profit. In addition, the study delves into the quantum game equilibrium of decentralized decision-making, resulting in the attainment of a carbon level and price that outperforms those derived from classical game theory.

The symbols utilized in this article and their respective meanings are detailed in Table 1.

**Table 1:** Definition of symbols.

Symbol	Definition
m, r	Manufacturer, retailer;
c	The cost of the product;
$p_{m}$, $p_{r}$	Online sales price, offline retail price;
$D_{m}$, $D_{r}$	Consumer’s demand of Online channel, consumer’s demand of offline channel;
k	Consumers’ Low-carbon preference;
β	Cross price elasticity coefficient of the offline channel and online channel;
e	Low-carbon level of products;
α	The maximum possible price of the product in the market;
w	Wholesale price.

The dominating element in this game is the manufacturer, whose dominance is a pivotal factor. In order to ensure the concavity of the profit function for each subject, the model operates under the assumption of a feasible public domain $k^{2}<1-\beta$. The demand functions for both the offline and online channels are as follows.


$$D_{r}=\alpha -p_{r}+ke+\beta p_{m}$$ (1)

$$D_{m}=\alpha -p_{m}+ke+\beta p_{r}$$ (2)

Drawing from the literature of Yang et al. [44], it is posited that the cost of efforts aimed at enhancing the Low-carbon level of products is a quadratic function of the Low-carbon level of products $\frac{1}{2}e^{2}$. In the following research, the superscripts “*C*,” “*N*,” and “*Q*” are employed to denote the strategies of centralized decision-making, decentralized decision-making, and decentralized decision-making under the quantum game, respectively.

### 3.1. Classical Game Models

#### 3.1.1. Centralized decision model.

According to the problem description, fundamental assumptions, and demand function, the profit function of the supply chain is as follows:


$$\pi^{C}=p_{r}(\alpha -p_{r}+ke+\beta p_{m})+(p_{m}-c)(\alpha -p_{m}+ke+\beta p_{r})-\frac{1}{2}e^{2}$$ (3)

It can be verified by Hesser matrix with respect to *e*, $p_{r}$ and $p_{m}$ according to profit function $\pi^{C}$ that the profit maximization of dual-channel supply chain exists and is unique. By calculating the mixed partial derivatives, we can obtain the following matrix:


$$H^{C}=\left[\begin{array}{ccc} {}*20c-1 & k & k\\ k & -2 & 2\beta \\ k & 2\beta & -2 \end{array}\right]$$ (4)

The First-order sequential master subexpression $H_{1}^{C}=-1$, the Second-order sequential master subexpression $H_{2}^{C}=2-k^{2}$, and the Third-order sequential master subexpression $H_{3}^{C}=4(1+\beta )(k^{2}+\beta -1)$ of the matrix $H^{C}$ can be obtained. In order for $\pi^{C}$ to be a joint concave function on *e*, *p*_*r*_ and *p*_*m*_, $H_{1}^{C}<0$, $H_{2}^{C}>0$ and $H_{3}^{C}<0$ must be satisfied at the same time. Therefore, we obtain *k*^2^ < 1 ‒ *β* such that *π*^*C*^ is joint concave on *e*, *p*_*r*_ and *p*_*m*_. It indicates that under centralized decision-making, the maximum profit of a dual-channel supply chain exists and is unique.

Therefore, let the first derivatives of $\pi^{C}$ with respect to *e*, *p*_*r*_ and *p*_*m*_ be 0, $\frac{\partial \pi^{C}}{\partial e}=0$, $\frac{\partial \pi^{C}}{\partial p_{r}}=0$ and $\frac{\partial \pi^{C}}{\partial p_{m}}=0$, respectively. The aforementioned formulas are collectively solved, yielding the optimal Low-carbon level, online sales price, and offline sales price for retailers under centralized decision-making, thereby maximizing the profits of the two suppliers.


$$e^{C*}=\frac{k(2\alpha +c\beta -c)}{2\left(1-k^{2}-\beta \right)}$$ (5)

$$p_{m}^{C*}=\frac{2\alpha -(3k^{2}+2\beta -2)c}{4\left(1-k^{2}-\beta \right)}$$ (6)

$$p_{r}^{C*}=\frac{2\alpha -ck^{2}}{4\left(1-k^{2}-\beta \right)}$$ (7)

At this time, the optimal profit of the supply chain is:


$$\pi^{C*}=\frac{4\alpha^{2}-\left(\beta k^{2}+k^{2}+2\beta -2\right)c^{2}+4\alpha (\beta -1)c}{8(1-k^{2}-\beta )}$$ (8)

**Proposition 1** In a centralized decision mode, (1) $p_{m}^{C*}>p_{r}^{C*}$; (2) $e^{C*}$, $p_{m}^{C*}$, $p_{r}^{C*}$ and $\pi^{C*}$ increase in β; (3) $p_{m}^{C*}$, $p_{r}^{C*}$, $\pi^{C*}$ and $e^{C*}$ increase in *k*.

In the context of centralized decision-making, it is customary for online and offline channels to employ differentiated pricing strategies. This discrepancy arises from cost differentials, leading to the optimal pricing decision wherein the sales price in the online channel surpasses that of the offline channel, thereby maximizing the overall profit in the supply chain. In the framework of a dual-channel supply chain, the increasing consumer preference for low-carbon products, coupled with greater market competition, serves to elevate the low-carbon attributes of products. Therefore, manufacturers are compelled to intensify their efforts in low-carbon research and development, thereby enhancing the low-carbon and eco-friendly nature of their products. This increased effort translates to a corresponding increase in the prices of direct online sales and offline retail channels, thus stimulating demand and enhancing the total profit of the supply chain. The post-price increase demand surpasses the pre-increase levels, primarily due to the more pronounced positive influence of the low-carbon attributes on demand, outweighing the negative impact of the price hike.

#### 3.1.2. Decentralized decision model.

Conversely, under a decentralized decision-making paradigm, manufacturers are driven by the necessity to maximize their individual interests. Firstly, manufacturers determine the low-carbon attributes of their products, the wholesale prices from suppliers, and the sales prices in online channels. Subsequently, retailers decide the offline retail prices of products with the aim of profit maximization. Upon revisiting the model description, the profit function of retailers and manufacturers is as follows:


$$\pi_{r}^{N}=(p_{r}-w)(\alpha -p_{r}+ke+\beta p_{m})$$ (9)

$$\pi_{m}^{N}=(p_{m}-c)(\alpha -p_{m}+ke+\beta p_{r})+w(\alpha -p_{r}+ke+\beta p_{m})-\frac{1}{2}e^{2}$$ (10)

According to the inverse solution method, starting from the profit function of retailers, the second derivative of the Equation (2) with respect to $p_{r}$, $\frac{\partial^{2}\pi_{r}^{N}}{\partial^{2}p_{r}}=-2$, it shows that $\pi_{r}^{N}$ is a concave function with respect to $p_{r}$ and has a unique maximum value. The first derivative of $\pi_{r}^{N}$, with respect to $p_{r}$, $\frac{\partial \pi_{r}^{N}}{\partial p_{r}}=0$, is obtained $p_{r}(e,w,p_{m})=\frac{\alpha +w+ke+\beta p_{m}}{2}$. After putting $p_{r}(e,w,p_{m})$ into the Equation (3), the Hessian matrix of $\pi_{m}^{N}$ with respect to *e*, *w* and $p_{m}$ is obtained:


$$H^{N}=\left[\begin{array}{ccc} {}*20c-1 & \frac{k}{2} & k+\frac{\beta k}{2}\\ \frac{k}{2} & -1 & \beta \\ k+\frac{\beta k}{2} & \beta & -2 \end{array}\right]$$ (11)

According to $H^{N}$, there exists a unique maximum solution for the manufacturer’s profit $\pi_{m}^{N}$ at $0<k^{2}<\frac{4(1-\beta )}{3+\beta }$. Take the first derivatives of $\pi_{m}^{N}$ with respect to *e*, *w* and $p_{m}$, $\frac{\partial \pi^{C}}{\partial e}=0$, $\frac{\partial \pi^{C}}{\partial w}=0$ and $\frac{\partial \pi^{C}}{\partial p_{m}}=0$, and solve them jointly to obtain the optimal low-carbon level, wholesale price, and online sale price that maximizes the manufacturer’s profit under decentralized decision-making:


$$w^{N*}=\frac{4\alpha -(\beta +2)k^{2}c}{2\left(4(1-\beta )-(3+\beta )k^{2}\right)}$$ (12)

$$e^{N*}=k\frac{(3+\beta )\alpha -(1-\beta )(\beta +2)c}{4(1-\beta )-(3+\beta )k^{2}}$$ (13)

$$p_{m}^{N*}=\frac{4\alpha -\left(\left(2\beta +5)k^{2}-4(1-\beta \right)\right)c}{2\left(4(1-\beta )-(3+\beta )k^{2}\right)}$$ (14)

Where $4\alpha -\left(\left(2\beta +5)k^{2}+4(\beta -1\right)\right)c>4\alpha -(\beta +2)k^{2}c>(3+\beta alpha-(1-\beta )(\beta +2)c>0$, so $\alpha >\frac{(1-\beta )(\beta +2)c}{\left(3+\beta \right)}$. Put $w^{N*}$, $e^{N*}$, and $p_{m}^{N*}$ into $p_{r}(e,w,p_{m})$ to get the offline sale price that profit maximization the retailer:


$$p_{r}^{N*}=\frac{2(3-\beta )\alpha -(2\beta^{2}-2\beta +2\beta k^{2}+3k^{2})c}{2\left(4(1-\beta )-(3+\beta )k^{2}\right)}$$ (15)

At this time, the maximum profits of manufacturers and retailers are:


$$\pi_{r}^{N*}=\frac{{\left(2\beta^{2}c+\left(ck^{2}+2\alpha -2c\right)\beta +ck^{2}-2\alpha \right)}^{2}}{4{\left(\beta k^{2}+3k^{2}+4\beta -4\right)}^{2}}$$ (16)

$$\pi_{m}^{N*}=\frac{2(3+\beta )\alpha^{2}+4(\beta^{2}+\beta -2)\alpha c+\left(2(2-\beta^{2})(1-\beta )-(\beta +1)k^{2}\right)c^{2}}{4\left(4(1-\beta )-(\beta +3)k^{2}\right)}$$ (17)

**Proposition 2:** In the decentralized decision mode, (1) $p_{m}^{C*}<p_{r}^{C*}$; (2) $w^{N*}$, $e^{N*}$, $p_{m}^{N*}$, $p_{r}^{N*}$, $\pi_{m}^{N*}$ and $\pi_{r}^{N*}$ increase in k; (3) $w^{N*}$, $e^{N*}$, $p_{m}^{N*}$, $p_{r}^{N*}$, $\pi_{m}^{N*}$ and $\pi_{r}^{N*}$ increase in β.

In the decentralized decision-making process, significant fluctuations occur in the sales prices of products across various sales channels. This phenomenon arises from the higher operational costs associated with offline sales in comparison to online sales, leading to a customary elevation of offline sales prices to bolster operational profits. As market competition intensifies, the wholesale costs, low-carbon thresholds, and sales prices in online channels also experience an upsurge. Correspondingly, the sales prices in offline channels witness a similar increase, thereby augmenting the optimal profits for manufacturers and retailers alike. Nevertheless, as manufacturers are able to derive revenue from both channels, whereas retailers solely accrue profits from retail channels, the latter’s profit margin remains inferior to that of the former. In the decentralized decision-making framework, the enhancement of consumers’ low-carbon consciousness also contributes to the enhancement of optimal profits. Owing to the influence of pricing dynamics and shopping convenience, an increasing number of consumers opt to procure low-carbon products through online direct marketing channels.

### 3.2. Quantum game model

#### 3.2.1. Quantum model description.

The diverse attributes inherent in quantum theory are particularly well-suited for explaining the intricacies of the information industry. This theory posits that the external “existence of the world” is complicatedly interwoven with our perceptual faculties. Piotrowski and Sadkowski’s quantum game methodology offers a viable means to explicate the information exchange dynamics in markets [45]. In this framework, a quantum strategy assumes the form of a vector in a Hilbert space, which can be construed as a superposition of strategic choices. From the perspective of decision-makers, their strategies embody “quantum bits,” and the subject of an actual decision may consist of a single decision-maker, a decision-making consortium, or even the entire market.At the core of this theoretical framework lies the concept of strategy. Whether arising spontaneously or as a result of institutionalized market transactions, these actions are conceptualized as projective operations in a Hilbert space, exerting influence upon the decision maker’s strategy, which is in turn represented by the probability amplitude (wave function) |φ⟩.

Quantum entanglement emerges as a requisite condition for the attainment of strategic equilibrium, it usually refers to the phenomenon that multiple microscopic particles intertwine with each other due to the characteristics of the microscopic system. Quantum entanglement represents the non-localized, non-classical decision correlation based on public information among decision makers in market competition. For example, Volkswagen and Ford, which compete fiercely in the global electric vehicle market, are also working together to develop an electric vehicle platform. Quantum entanglement in quantum game theory can be used to describe the complex competitive relationship between Volkswagen and Ford in the low-carbon technology and electric vehicle markets. In the competition, Volkswagen and Ford’s decisions affect each other, such as adjusting pricing and technology strategy; In the collaboration, the two companies’ joint research and development on the electric vehicle platform makes their resources and technology closely linked, and the decisions of either will affect the other. The quantum game model can help analyze this complex competition and cooperation relationship, so as to optimize their decisions on low-carbon emission reduction and market strategies.

In classical information processing, information is encoded using classical binary bits, each capable of existing in one of two states (1 and 0). The Hilbert space generated by the base vector is termed a qubit, and its state may assume a superposition of multiple states concurrently. Herein, two market participants each possess a qubit and are empowered to execute quantum operations on their respective qubits. The tensor product of qubits can represent the state of the game at each moment, assuming that the game between manufacturer and retailer starts at |vac1⟩⊗|vac2⟩. The first and second terms pertain to the qubits held by the manufacturer and retailer, respectively. The initial state is transformed into a quantum entanglement state $\left|\phi_{0}\right\rangle =\hat{J}(\gamma )(\left|vac_{1}\right\rangle ⊗\left|vac_{2}\right\rangle )$ by a particular unitary operator $\hat{J}(\gamma )$. The unitary entanglement operator $\hat{J}$ is the common knowledge of both sides of the enterprises, where γ≥0 denotes the compression parameter or degree of entanglement. The entangled system defies description of the entire quantum system by independently characterizing the state of each qubit. The state of the two enterprise subsystems is interdependent, with each existing in an uncertain state. The connection between them transcends temporal and spatial considerations. Subsequently, this state is communicated to each participant, empowering them to select their individual strategies.

The action strategy can be expressed through the utilization of unitary operators ${\hat{U}}_{0}$ and ${\hat{U}}_{1}$, which signify the localized operators acting upon their respective magnetic fields, strategy selection, and a specific array of unitary operators: ${\hat{U}}_{j}(x_{j})=\exp(-ix_{j}{\hat{P}}_{j})$. Where ${\hat{P}}_{j}=\frac{i}{\sqrt{2}}({\hat{a}}_{j}^{+}-{\hat{a}}_{j}),j=0,1$. Operators ${\hat{a}}_{j}$ and ${\hat{a}}_{j}^{+}$ are the annihilation and generation operators of the j-mode optical, electromagnetic field. Subsequently, the two entities are conveyed through the measurement apparatus subsequent to the action of the disentanglement operator ${\hat{J}}^{\mathrm{\backslash dag}}$ on the measuring state, thereby the final state of the game measurement is $\left|\phi_{f}\right\rangle =\hat{J}(\gamma {)}^{\mathrm{\backslash dag}}({\hat{U}}_{0}⊗{\hat{U}}_{1}hatJ(\gamma )(\left|vac1\right\rangle ⊗\left|vac2\right\rangle )$. The fundamental form of the entanglement operator is $\hat{J}(\gamma )=\exp\{-\gamma ({\hat{a}}_{1}^{\mathrm{\backslash dag}}{\hat{a}}_{2}^{\mathrm{\backslash dag}}-{\hat{a}}_{1}{\hat{a}}_{2}\}$. In order to maintain the symmetry of the game, $\hat{J}(\gamma )$ is also symmetric. Following the measurement, the result corresponds to the eigenstate of the final coherence state. The price tags of the two entities are as follows:


$$p_{r}^{Q}(x_{1},x_{2})=x_{r}\cosh\gamma +x_{m}\sinh\gamma$$ (18)

$$p_{m}^{Q}(x_{1},x_{2})=x_{r}\sinh\gamma +x_{m}\cosh\gamma$$ (19)

Where, γ denotes the entanglement degree of the quantum game, $\sinh\gamma =(e^{\gamma }-e^{-\gamma })/2$, and $\cosh\gamma =(e^{\gamma }+e^{-\gamma })/2$. $x_{r}$ and $x_{m}$ represent the strategies used by manufacture and retailer. When γ=0, namely $\hat{J}(\gamma )=I$, the entanglement operator is the identity matrix, $x_{r}$ and $x_{m}$ represent the output under the classical model, $p_{r}=x_{r}$ and $p_{m}=x_{m}$.

#### 3.2.2. Quantum game equilibrium.

By put Equations (18) and (19) into Equations (1) and (2), the retailer’s profit can be written as $\pi_{r}=\left(x_{r}\cosh\gamma +x_{m}\sinh\gamma -w\mathrm{\backslash rightleft}(\alpha +(\beta x_{m}-x_{r})\cosh\gamma +(\beta x_{r}-x_{m})\sinh\gamma +ke\right)$. According to the Hesser matrix of the profit function and Klem’s rule, the first derivative condition $\frac{\partial \pi_{r}}{\partial x_{r}}=0$ of the retailer’s profit function can be obtained:


$$x_{r}=\frac{\beta x_{m}(\cosh^{2}\gamma +\sinh^{2}\gamma )-2x_{m}\cosh\gamma \sinh\gamma +(ek+\alpha +w)\cosh\gamma -\beta w\sinh\gamma }{2\cosh\gamma (-\beta \sinh\gamma +\cosh\gamma )}$$ (20)

Putting the above equation into the manufacturer profit Equations (9), we can obtain the manufacturer’s decision variables $x_{m}^{0}$, Low-carbon effort level $e^{Q*}$ and wholesale price $w^{Q*}$ according to the first derivative conditions $\frac{\partial \pi_{m}}{\partial x_{m}}=0$, $\frac{\partial \pi_{m}}{\partial e}=0$, $\frac{\partial \pi_{m}}{\partial w}=0$ of the manufacturer profit function,


$$x_{m}^{0}=\frac{\left(\begin{array}{c} 2\left(\left(3ck^{2}-2\alpha +4c\right)\beta +5ck^{2}-4c-4\alpha \right)\cosh^{2}\gamma \\ +\left(6\alpha -\left(5k^{2}-4+4\beta \right)\beta c-3ck^{2}\right)\sinh2\gamma +2\left(2\alpha -ck^{2}\right)\beta \end{array}\right)}{2\left(2\beta k^{2}+6k^{2}+8\beta -8\right)\cosh\gamma -4\beta \sinh\gamma \left(k^{2}+\beta -1\right)}$$ (21)

$$e^{Q*}=-k\frac{\left((\beta +3)\cosh\gamma -2\beta \sinh\gamma \right)\alpha +\left(\left(\beta^{2}+\beta -2\right)\cosh\gamma +\beta \left(1-\beta \right)\sinh\gamma \right)c}{\left(\left(\beta +3)k^{2}+4(\beta -1\right)\right)\cosh\gamma +2\beta \left(1-k^{2}-\beta \right)\sinh\gamma }$$ (22)

$$w^{Q*}=-\frac{\left(-2\beta \sinh\gamma +4\cosh\gamma \right)\alpha +\left(\beta \sinh\gamma -(\beta +2)\cosh\gamma \right)k^{2}c}{2\left(\left(\beta +3)k^{2}+4(\beta -1\right)\right)\cosh\gamma +4\beta \left(1-k^{2}-\beta \right)\sinh\gamma }$$ (23)

Put $x_{m}^{0}$, $e^{Q*}$, and $w^{Q*}$ into $x_{r}$, we can obtain:


$$x_{r}^{0}=\frac{\left(\begin{array}{c} 2\left(\left(5k^{2}-4+4\beta \right)\beta c+3ck^{2}-6\alpha \right)\cosh^{2}\gamma -2\left(3ck^{2}-2(\alpha +c-c\beta )\right)\beta -\\ \left(\left(3ck^{2}+4c-2\alpha \right)\beta +5ck^{2}-4(\alpha +c)\right)\sinh2\gamma \end{array}\right)}{2\left(2\beta k^{2}+6k^{2}+8\beta -8\right)\cosh\gamma -4\beta \left(k^{2}+\beta -1\right)\sinh\gamma }$$ (24)

Putting $x_{m}^{0}$, $e^{Q*}$, $w^{Q*}$ and $x_{r}^{0}$ into Equations (25) (6), (7) and Equations (3), (4) respectively. The optimal price $p_{m}^{Q*}$ and $p_{r}^{Q*}$ of manufacturer and retailer can be obtained:


$$p_{r}^{Q*}=\frac{\left(2\beta^{2}c+\left(2ck^{2}+2\alpha -2c\right)\beta +3ck^{2}-6\alpha \right)\cosh\gamma -\beta \left(ck^{2}-2\alpha \right)\sinh\gamma }{\left(2\beta k^{2}+6k^{2}+8\beta -8\right)\cosh\gamma -4\beta \left(k^{2}+\beta -1\right)\sinh\gamma }$$ (25)

$$p_{m}^{Q*}=\frac{\left(\left(2k^{2}+4\right)c\beta +\left(5k^{2}-4\right)c-4\alpha \right)\cosh\gamma -\sinh\gamma \left(\left(3k^{2}+2\beta -2\right)c-2\alpha \right)\beta }{\left(2\beta k^{2}+6k^{2}+8\beta -8\right)\cosh\gamma -4\beta \sinh\gamma \left(k^{2}+\beta -1\right)}$$ (26)

At this time, the optimal profit of the manufacturer and retailer is:


$$\pi_{m}^{Q*}=\frac{\left(\begin{array}{c} \left(\left(\left(1-\beta \right)2\beta^{2}+\left(k^{2}+4\right)\left(\beta +1\right)\right)c^{2}-4\alpha \left(\beta +2\right)\left(\beta -1\right)c-2\alpha^{2}\left(\beta +3\right)\right)\cosh\gamma \\ -\beta \left(\left(\beta +1\right)c^{2}k^{2}+(\beta -1)\left(2c+4\alpha \right)c-4\alpha^{2}\right)\sinh\gamma \end{array}\right)}{4\left(\beta k^{2}+3k^{2}+4\beta -4\right)\cosh\gamma -8\beta \left(k^{2}+\beta -1\right)\sinh\gamma }$$ (27)

$$\pi_{m}^{Q*}=\frac{\left(\begin{array}{c} \left(\left(k^{2}-4\right)c^{2}+8\alpha c-6\alpha^{2}\right)\cosh\gamma \\ +\left(-2\beta^{3}c^{2}+2(c-2\alpha )c\beta^{2}+\left(\left(k^{2}+4\right)c^{2}-4\alpha c-2\alpha^{2}\right)\beta \right)\cosh\gamma \\ -\beta \left(\left(\beta +1\right)c^{2}k^{2}+(\beta -1)\left(2c+4\alpha \right)c-4\alpha^{2}\right)\sinh\gamma \end{array}\right)}{4\left(\beta k^{2}+3k^{2}+4\beta -4\right)\cosh\gamma -8\beta \left(k^{2}+\beta -1\right)\sinh\gamma }$$ (28)

$$\pi_{r}^{Q*}=\frac{-\cosh\gamma \left(\beta \sinh\gamma -\cosh\gamma \right){\left(2\beta^{2}c+\left(ck^{2}+2\alpha -2c\right)\beta +ck^{2}-2\alpha \right)}^{2}}{4\left(\begin{array}{c} \left(4\beta^{4}+8\left(k^{2}-1\right)\beta^{3}+5\left(k^{2}+4\right)\beta^{2}\right)\cosh^{2}\gamma +\\ \left(2\left(3k^{4}+8k^{2}-16\right)\beta +3{\left(3k^{2}-4\right)}^{2}\right)\cosh^{2}\gamma -\\ 2\beta \left(k^{2}+\beta -1\right)\left((\beta +3)k^{2}+4\beta -4\right)\sinh2\gamma -4\beta^{2}{\left(k^{2}+\beta -1\right)}^{2} \end{array}\right)}$$ (29)

**Proposition 3:**

(1) When γ→0, then


$$\underset{\gamma \to 0}{\lim}e^{Q*}=k\frac{(3+\beta )\alpha -(1-\beta )(\beta +2)c}{4(1-\beta )-(3+\beta )k^{2}},$$

$$\underset{\gamma \to 0}{\lim}p_{r}^{Q*}=\frac{2(3-\beta )\alpha -(2\beta^{2}-2\beta +2\beta k^{2}+3k^{2})c}{2\left(4(1-\beta )-(3+\beta )k^{2}\right)},$$

$$\underset{\gamma \to 0}{\lim}w^{Q*}=\frac{4\alpha -(\beta +2)k^{2}c}{2\left(4(1-\beta )-(3+\beta )k^{2}\right)},$$

$$\underset{\gamma \to 0}{\lim}\pi_{r}^{Q*}=\frac{{\left(2\beta^{2}c+\left(ck^{2}+2\alpha -2c\right)\beta +ck^{2}-2\alpha \right)}^{2}}{4{\left(\beta k^{2}+3k^{2}+4\beta -4\right)}^{2}},$$

$$\underset{\gamma \to 0}{\lim}p_{m}^{Q*}=\frac{4\alpha -\left(\left(2\beta +5)k^{2}-4(1-\beta \right)\right)c}{2\left(4(1-\beta )-(3+\beta )k^{2}\right)},$$

and


$$\underset{\gamma \to 0}{\lim}\pi_{m}^{Q*}=\frac{2(3+\beta )\alpha^{2}+4(\beta^{2}+\beta -2)\alpha c+\left(2(2-\beta^{2})(1-\beta )-(\beta +1)k^{2}\right)c^{2}}{4\left(4(1-\beta )-(\beta +3)k^{2}\right)}$$

(2) When γ→∞, then


$$\underset{r\to \infty }{\lim}e^{Q*}=\frac{(3-\beta )\alpha +2(\beta -1)c}{\left(\beta -3)k^{2}+2(\beta^{2}-3\beta +2\right)}k,$$

$$\underset{r\to \infty }{\lim}w^{Q*}=\frac{-ck^{2}-\alpha \beta +2\alpha }{\beta k^{2}+2\beta^{2}-3k^{2}-6\beta +4},$$

$$\underset{\gamma \to \infty }{\lim}p_{m}^{Q*}=\frac{\beta ck^{2}+2\beta^{2}c-5ck^{2}-2\alpha \beta -6\beta c+4\alpha +4c}{2\beta k^{2}+4\beta^{2}-6k^{2}-12\beta +8},$$

$$\underset{\gamma \to \infty }{\lim}p_{r}^{Q*}=\frac{-\beta ck^{2}-2\beta^{2}c-3ck^{2}-4\alpha \beta +2\beta c+6\alpha }{2\beta k^{2}+4\beta^{2}-6k^{2}-12\beta +8},$$

$$\underset{\gamma \to \infty }{\lim}\pi_{m}^{Q*}=\frac{\beta^{2}c^{2}k^{2}+2\beta^{3}c^{2}-c^{2}k^{2}-2\alpha^{2}\beta +8\alpha \beta c-6\beta c^{2}+6\alpha^{2}-8\alpha c+4c^{2}}{4\beta k^{2}+8\beta^{2}-12k^{2}-24\beta +16},$$

and


$$\underset{\gamma \to \infty }{\lim}\pi_{r}^{Q*}=-\frac{{\left(2\beta^{2}c+\left(ck^{2}+2\alpha -2c\right)\beta +ck^{2}-2\alpha \right)}^{2}(\beta -1)}{4{\left(\beta k^{2}+2\beta^{2}-3k^{2}-6\beta +4\right)}^{2}}$$

As the entanglement approaches 0, the result converges between the quantum game equilibrium and the Nash equilibrium, while still differing from the centralized decision. Conversely, as the entanglement tends towards infinity, the quantum game equilibria and Nash equilibria yield different results. Therefore, the quantum game has the capacity to change the decision state of game participants and generate results different from those of classical games.

**Corollary 1**: In quantum games, (1) $e^{Q*}$ increase in γ, $w^{Q*}$ and $p_{m}^{Q*}$ decrease in γ; (2) if $k^{2}+\frac{2\beta }{3}>\frac{2}{3}$, then $p_{r}^{Q*}$ decrease in γ; if $k^{2}+\frac{2\beta }{3}<\frac{2}{3}$, then $p_{r}^{Q*}$ increase in γ.

In the quantum game mode, the manufacturer’s optimal decision variables $w^{Q*}$, $e^{Q*}$ and $p_{m}^{N*}$ all exhibited a monotonic correlation with the level of entanglement in the quantum game. This correlation was found to be independent of both the consumers’ low-carbon preference *k* and market competition intensity β. Specifically, as the entanglement between enterprises increased, the low-carbon level of the product also increased. Simultaneously, due to the rise in costs, in order to attract more consumers to buy products, both the wholesale price of the product and the sales price of online direct sales channels have to experience a downward trend. The optimal offline retail price $p_{r}^{Q*}$ changes in response to the increase in entanglement, and its fluctuation was contingent upon the consumers’ low-carbon preference *k* and market competition intensity β. When the low-carbon preference of consumers and the fierce market competition reached a certain threshold, the impact of entanglement between enterprises on the retail price mirrored that of the online direct marketing channel price. Conversely, if the low-carbon preference of consumers was insufficient and the market competition was not intense, the entanglement between enterprises would contribute to a reduction in the optimal price of retailers.

## 4. Analysis of manufacturer’s optimal decision

To further understand the impact of quantum games on manufacturers’ optimal decisions, the Optimal low-carbon level, Optimal price and Optimal profit of the manufacturer are analyzed in different entanglement cases. We compare and prove the three decision models, namely the centralized decision (*C*), decentralized decision (Nash equilibrium (*N*)), and quantum game equilibrium (*Q*).

### 4.1. Optimal decision analysis

#### 4.1.1. Optimal low-carbon level.

According to the equilibrium income of the manufacturer in Section 3, the change of the manufacturer’s optimal Low-carbon level in the quantum strategy is analyzed. This examines the impact of entanglement on manufacturers’ decisions on carbon reduction efforts.

**Proposition 4:** (1) $e^{Q*}\geq e^{N*}>0$, no matter how γ changes; (2) $e^{C*}>e^{Q*}$. (3) when γ→0, then $e^{C*}>e^{Q*}=e^{N*}$; when γ→∞, then $e^{Q*}>e^{C*}>e^{N*}$.

In the analysis of manufacturers’ decision-making processes, the impact of quantum entanglement on the low-carbon level is analyzed. When the interconnection among enterprises is 0, the low-carbon level in quantum decision-making aligns with that of decentralized decision-making. However, as the entanglement between enterprises intensifies, the low-carbon level in quantum decision-making consistently surpasses that of decentralized decision-making. Notably, the low-carbon level in centralized decision-making exceeds that of quantum decision-making. Nevertheless, as the entanglement approaches infinity, the low-carbon level in quantum decision-making surpasses that of centralized decision-making, irrespective of consumers’ low-carbon preferences and market competition intensity. Evidently, quantum decision entanglement effectively enhances the low-carbon level of manufacturers’ products.

#### 4.1.2. Optimal price.

Price is a direct manifestation of the effect of quantum entanglement, which leads to changes in firms’ decision. The change of the firms’ optimal price in the quantum strategy is analyzed. This examines the impact of entanglement on firms’ decisions on price.

**Proposition 5:** (1) $w^{Q*}<w^{N*}$ and $p_{m}^{Q*}<p_{m}^{N*}$, no matter how γ changes. If $k^{2}+\frac{2\beta }{3}>\frac{2}{3}$, then $p_{r}^{Q*}<p_{r}^{N*}$, otherwise $p_{r}^{Q*}>p_{r}^{N*}$. (2) $p_{m}^{Q*}<p_{m}^{C*}$. If $k^{2}+\frac{2\beta }{3}>\frac{2}{3}$, then $p_{r}^{Q*}<p_{r}^{C*}$; otherwise $p_{r}^{Q*}>p_{r}^{C*}$. (3)when γ→0; then $w^{Q*}=w^{N*}$, $p_{m}^{Q*}=p_{m}^{N*}$, $p_{r}^{Q*}=p_{r}^{N*}$; when γ→∞, then $w^{Q*}>w^{N*}$ and $p_{m}^{Q*}>p_{m}^{C*}>p_{.m}^{N*}$. If $k^{2}+\frac{2\beta }{3}>\frac{2}{3}$, then $p_{r}^{Q*}>p_{r}^{C*}>p_{.r}^{N*}$, otherwise $p_{r}^{N*}>p_{r}^{C*}>p_{.r}^{Q*}$.

In Proposition 5, an analysis is conducted on the wholesale prices of manufacturers, the online sale prices, and the offline retail prices of retailers. As the degree of entanglement between enterprises approaches 0, the wholesale price, online sale price, and offline retail price under quantum decision align with the decentralized decision. However, as entanglement increases, the optimal wholesale price and online sale price of the manufacturer under quantum decision decrease less than that under decentralized decision. Notably, these results remain unaffected by the low-carbon preference of consumers and the intense market competition. The impact of quantum entanglement on the optimal offline retail price for retailers differs from the online sale price of the manufacturer. It is observed that only when consumers exhibit a high low-carbon preference and there is fierce market competition, can quantum decisions effectively reduce retailers’ optimal offline retail price, making it smaller than the optimal offline retail price under centralized and decentralized decisions. Otherwise, the optimal offline retail price under quantum decision surpasses that of the centralized and decentralized decisions.

Therefore, it is evident that the influence of quantum entanglement on the optimal decision of manufacturers is independent of consumers’ low-carbon preference and market competition. Quantum entanglement can notably reduce the optimal wholesale price and online sale price. Conversely, the impact of quantum entanglement on retailers’ optimal offline retail price is contingent upon the consumers’ low-carbon preference and market competition. This highlights that quantum entanglement exerts a more pronounced effect on manufacturers’ optimal pricing decisions than on retailers, and that retailers’ decisions are more attuned to consumers’ low-carbon preferences and the intensity of market competition than those of manufacturers.

#### 4.1.3. Optimal profit.

The change of profit directly reflects the result of the entanglement interaction between enterprises. Similarly, in this summary, we examine the impact of entanglement on firms’ decisions on optimal profit.

**Proposition 6:** (1) $\pi_{m}^{Q*}>\pi_{m}^{N*}$ and $\pi_{r}^{Q*}>\pi_{r}^{N*}$, no matter how γ changes. (2) $\pi_{m}^{Q*}+\pi_{r}^{Q*}<\pi^{C*}$. (3)when γ→0, then $\pi_{m}^{Q*}=\pi_{m}^{N*}$ and $\pi_{r}^{Q*}=\pi_{r}^{N*}$; when γ→∞, then $\pi_{m}^{Q*}>\pi_{m}^{N*}$ and $\pi_{r}^{Q*}>\pi_{r}^{N*}$, $\pi_{m}^{Q*}+\pi_{r}^{Q*}>\pi^{C*}$.

In Proposition 6, an analysis is conducted on the optimal profit of manufacturers and retailers in the context of quantum decision-making. When the entanglement factor is at 0, the optimal profit of both the manufacturer and the retailer in the quantum decision aligns with that of the decentralized decision. As the level of entanglement increases, the optimal profit of the manufacturer and retailer under a quantum decision surpasses that of a decentralized decision. This result remains unaffected by consumers’ low-carbon preferences and market competition. As the entanglement tends towards infinity, the optimal profit of the manufacturer and retailer under quantum decision exceeds that of the decentralized decision. In addition, the total profits of the two enterprises under the quantum decision surpass the total profit under the centralized decision.

### 4.2. Comparative analysis of game results

In this paper, the optimal decisions of manufacturer and retailer for the sale of green low-carbon products under different entanglement conditions are discussed, and the effects of entanglement contracts on the model’s decision parameters and optimal strategies are analyzed. In this section, we compare the game results of the firms in the quantum game and the classical game, as shown in Table 2.

**Table 2:** Comparison of manufacturer decision in classical case and quantum case.

Firms	parameter	Model comparison
manufacturer	e	$e^{C*}>e^{Q*}\geq e^{N*}>0$
	p	$w^{Q*}<w^{N*}$, $p_{m}^{Q*}\leq p_{m}^{N*}$, $p_{m}^{Q*}<p_{m}^{C*}$, $w^{Q*}>w^{N*}$, $p_{m}^{Q*}>p_{m}^{C*}>p_{.m}^{N*}$
	π	$\pi_{m}^{Q*}\geq \pi_{m}^{N*}$; $\pi_{m}^{Q*}+\pi_{r}^{Q*}<\pi^{C*}$
retailer	e	—
	p	$k^{2}+\frac{2\beta }{3}>\frac{2}{3}$, $p_{r}^{Q*}>p_{r}^{C*}>p_{.r}^{N*}$; $k^{2}+\frac{2\beta }{3}>\frac{2}{3}$, $p_{r}^{N*}>p_{r}^{C*}>p_{.r}^{Q*}$
	π	$\pi_{r}^{Q*}\geq \pi_{r}^{N*}$; $\pi_{m}^{Q*}+\pi_{r}^{Q*}<\pi^{C*}$

We compare carbon emission reduction levels, wholesale prices and profits of manufacturers under centralized decision making, decentralized decision making and quantum game models. The research results are similar to those of Ma et al. [46], Shi et al. [47] and Wang et al. [48], all of which show that centralized decision-making has the best performance in improving low-carbon effort level and corporate profits, while decentralized decision-making is relatively inefficient. However, this paper proposes a new decision-making framework by introducing a quantum game model, which further broadens the vision of low carbon supply chain management. The results show that the quantum game model not only better reveals the impact of information interaction on decision making among firms than the classical game model, but also improves low-carbon level and profits by adjusting entanglement, which has not been fully explored in the existing literature. Therefore, this paper provides a new theoretical perspective and practical path in the field of low-carbon supply chain management, with strong innovation and practicability.

As can be seen from Table 2, after manufacturers and retailers adopt quantum strategies, the low carbon level, equilibrium price and equilibrium profit all change. When the degree of entanglement increases in a quantum game:

(1) The optimal profits of both manufacturers and retailers have increased to varying degrees. Due to the increasing correlation of enterprise decision making, the increase (decrease) of the optimal equilibrium selling price has a greater impact on the optimal profit than the increase (decrease) of enterprise cost on the optimal equilibrium profit. Therefore, quantum game decision making can effectively increase the income of low-carbon production of strong enterprises. It is worth noting that when entanglement approaches infinity, the total profit of the two firms under quantum decisions exceeds the total profit under centralized decisions.
(2) Quantum decision-making improves the level of low-carbon efforts of enterprises. Due to the introduction of entanglement, the payoff state of an actor cannot be described independently, but is closely linked to the state of other actors. Manufacturers have the potential to achieve greater overall gains by increasing the level of low-carbon efforts in their businesses, rather than just consumers and other market participants getting the most out of it. This quantum synergy makes it possible for companies to increase the low-carbon level of their products no longer just a compromise, but an effective strategy to achieve common benefits.
(3) The impact of quantum entanglement on the optimal pricing decision of the manufacturer is more obvious than that of the retailer, whose decision is more adaptable to the low-carbon preference of consumers and the intensity of market competition than that of the manufacturer.

### 4.3. Numerical analysis

In sections 4.1 and 4.2, the influence of quantum entanglement on the optimal decision of manufacturers and retailers is analyzed respectively. In view of the complexity of the results, this section will use MATLAB analysis software and numerical simulation analysis method to conduct further analysis based on the conclusions obtained, and show the influence of entanglement on the production decision of low-carbon products in a more intuitive way. This section has representative and usable numerical examples that illustrate how an enterprise changes in an optimal decision situation with an entanglement of. By substituting the parameters into the analytic formula of the solution of the game model, the change trends of low-carbon level, price and profit under different decision models can be calculated respectively, and the results are analyzed as follows.

#### 4.3.1. Low-carbon level decision.

In this subsection, a numerical example is employed in this subsection to explicate the relationship between the low-carbon level of products on entanglement β and Low-carbon effort preference *k* and the intensity of market competition *β*. Representative yet hypothetical data are utilized. α=10,β=0.6,k=0.4,c=20.

**Fig 1:**
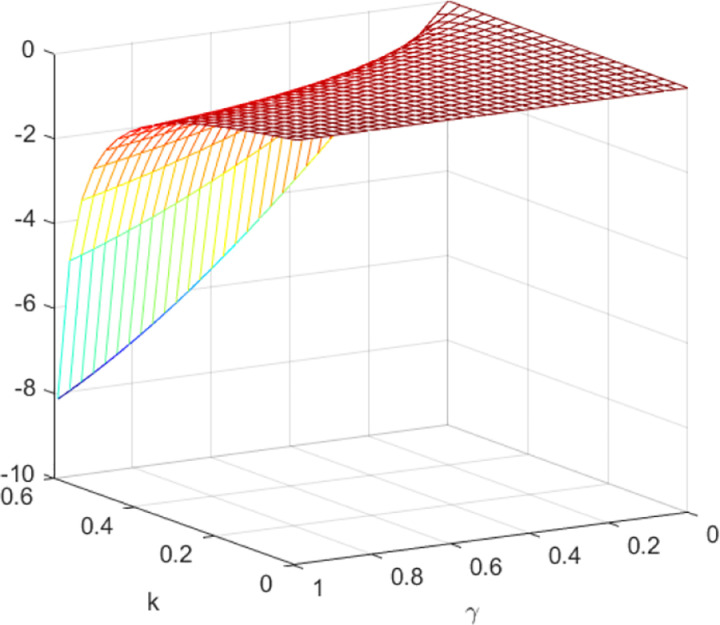
The impact of *γ* and *k* on $e^{N*}-e^{Q*}$.

**Fig 2:**
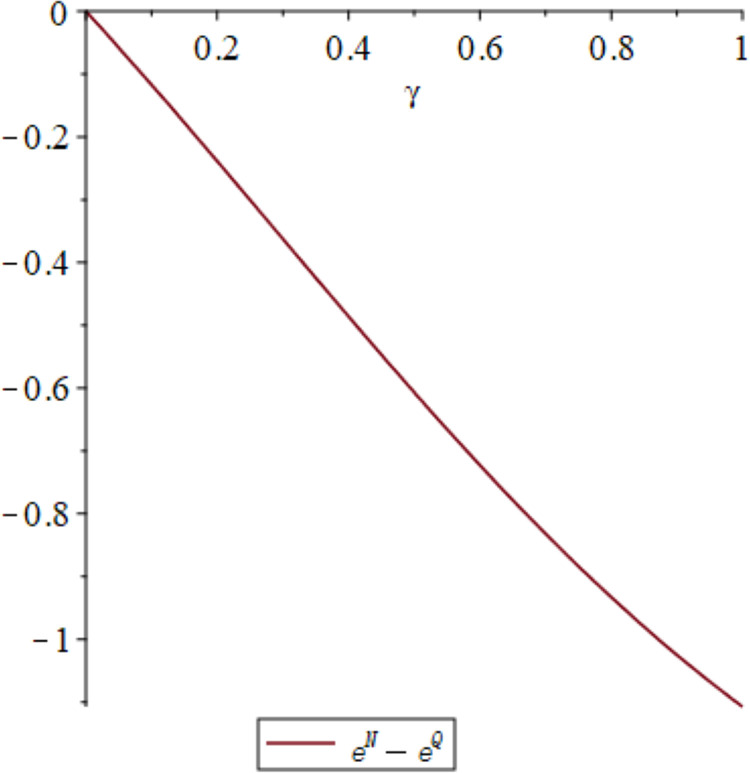
The impact of *γ* on $e^{N*}-e^{Q*}$ when *k* = 0.3.

The impact of quantum entanglement on the low-carbon level under classical decentralized and quantum decisions is explored, as depicted in Fig 1. According to the common picture usage habits of the paper [49], we have explained the pictures and listed the relevant parameters. Fig 1 shows the influence of parameters *γ* and *k* on variables $e^{N*}-e^{Q*}$, and shows the change trend and relationship of $e^{N*}-e^{Q*}$ under different values of *γ* and *k*. Irrespective of the level of entanglement, the low-carbon level under decentralized decision-making consistently lags behind that of quantum decision-making. Moreover, as entanglement intensifies, the difference between them widens. Fig 2 illustrates the sectional diagram of Fig 1 when *k* = 0.3, reinforcing the aforementioned conclusions. In addition, Fig 1 demonstrates that as consumers’ low-carbon preference increases, the superiority of the low-carbon level under quantum decision-making over classical decentralized decision-making becomes more pronounced.

**Fig 3:**
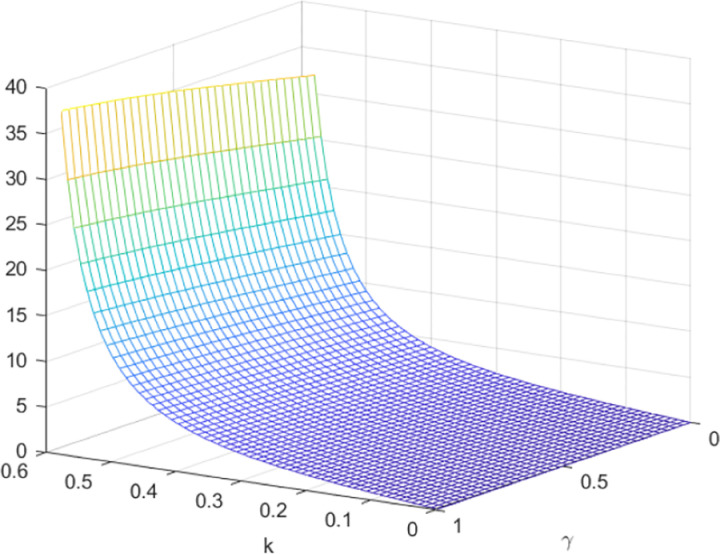
The impact of *γ* and *k* on $e^{C*}-e^{Q*}$.

Subsequently, numerical simulations are employed to visually represent the influence of quantum entanglement on low-carbon levels under centralized decision-making and quantum decision-making, as depicted in Fig 3. Fig 3 shows the influence of parameters *γ* and *k* on variables $e^{C*}-e^{Q*}$, and shows the change trend and relationship of $e^{C*}-e^{Q*}$ under different values of *γ* and *k*.

**Fig 4:**
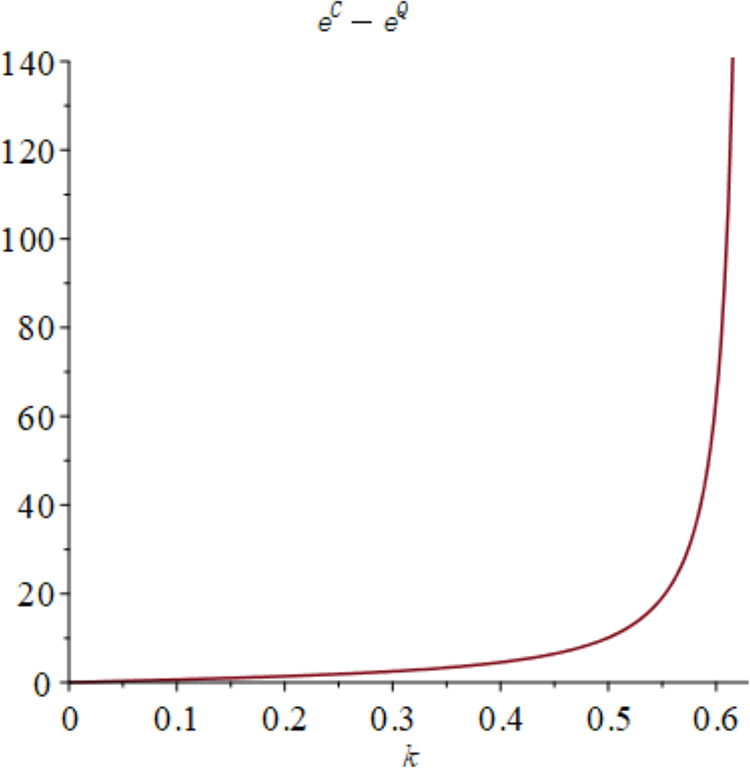
The impact of *k* on $e^{C*}-e^{Q*}$ when *γ*=0.4.

It is evident from the data presented in Fig 3 that the degree of entanglement among enterprises exerts minimal influence on the low-carbon level under both centralized decision-making and quantum decision-making. The disparity between these approaches is primarily dictated by the relationship between consumers’ inclination towards low-carbon options and the heightened intensity of market competition. Fig 4 illustrates the sectional diagram of Fig 3 for *γ*=0.4. When consumers exhibit a marginal preference for low-carbon alternatives, the low-carbon level resulting from centralized decision-making is mostly identical to that of quantum decision. As consumers’ proclivity for low-carbon options intensifies, the low-carbon level achieved through centralized decision-making consistently surpasses that of quantum decision-making. This gap widens in tandem with the degree of entanglement, signifying that quantum decision-making fails to enhance the low-carbon level relative to centralized decision-making.

As the degree of entanglement approaches infinity, the qualitative dynamics of the low-carbon level under centralized decision-making and quantum decision-making undergo a transformation. Under these circumstances, the low-carbon level resulting from quantum decision-making consistently surpasses that of centralized decision-making, irrespective of consumers’ low-carbon preferences and the intensity of market competition. It is evident that the optimal low-carbon level derived from the manufacturer’s decision-making process under quantum decision-making is responsive to consumers’ low-carbon preferences and the degree of entanglement. Quantum decision-making has the capacity to optimize the low-carbon level achieved through decentralized decision-making, and even outperforms the low-carbon level achieved through centralized decision-making when the degree of entanglement among enterprises approaches infinity.

#### 4.3.2. Price decision.

**Fig 5:**
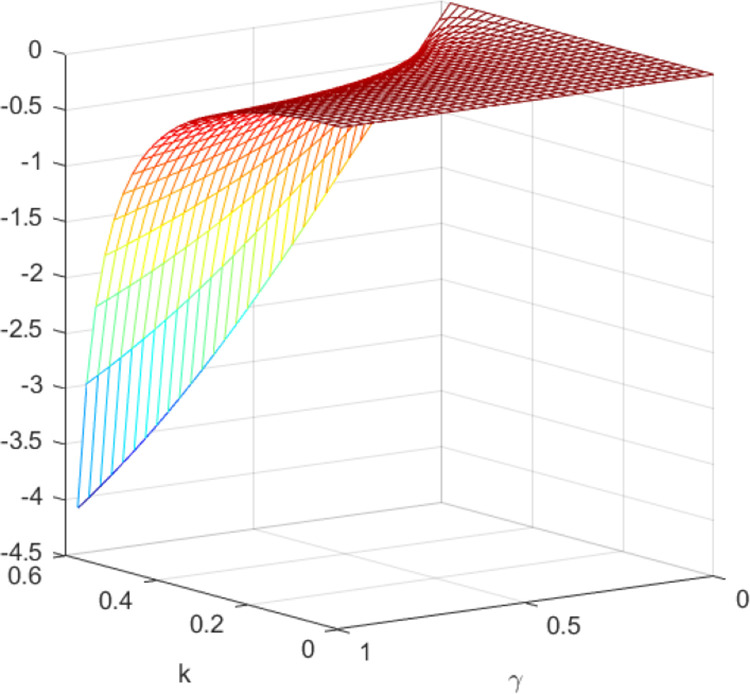
The impact of *γ* and *k* on. $w^{Q*}-w^{N*}$.

The influence of quantum entanglement on wholesale prices, online direct selling prices, and offline retail prices is demonstrated through the numerical simulation examples provided above. The influence of quantum entanglement on wholesale prices is analyzed in the context of classical decentralized decision-making and quantum decision-making, as depicted in Fig 5. Fig 5 shows the influence of parameters *γ* and *k* on variables $w^{Q*}-w^{N*}$, and shows the change trend and relationship of $w^{Q*}-w^{N*}$ under different values of *γ* and *k*.

**Fig 6:**
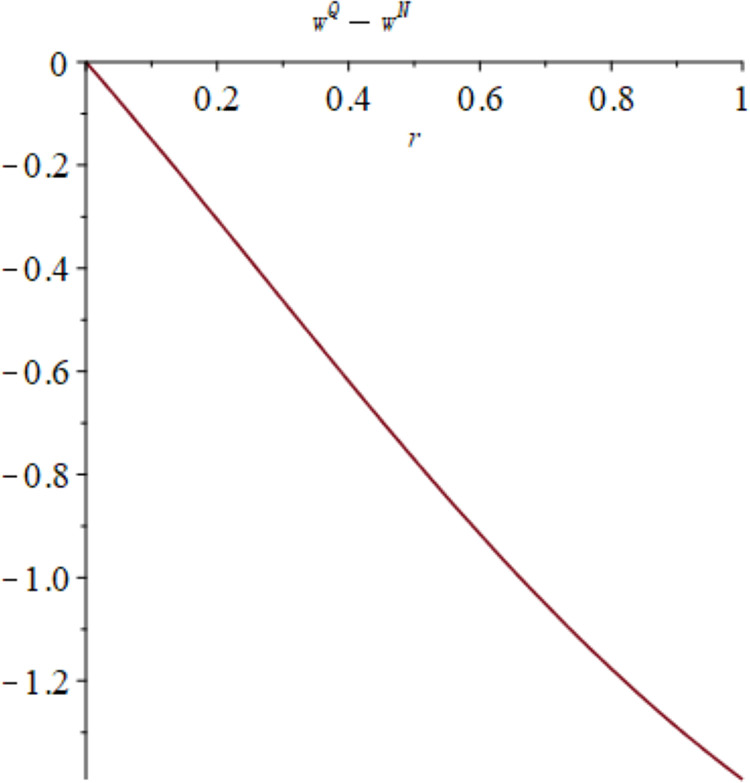
The impact of *γ* on $w^{Q*}-w^{N*}$ when *k* = 0.5.

It is observed that, irrespective of entanglement, the wholesale prices under decentralized decision-making consistently surpass those under quantum decision-making, with the difference between them widening as entanglement increases. Fig 6 illustrates the sectional diagram of Fig 5 for *k* = 0.5, reinforcing the aforementioned conclusions. In addition, as depicted in Fig 5, the influence of consumers’ low-carbon preference on wholesale prices accentuates the distinction between quantum decision-making and classical decentralized decision-making.

**Fig 7:**
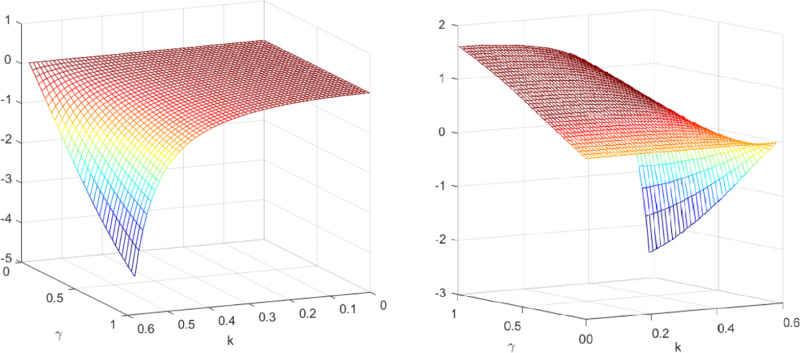
The impact of *γ* and *k* on $p_{m}^{Q*}-p_{m}^{N*}$ and. $p_{r}^{Q*}-p_{r}^{N*}$.

**Fig 8:**
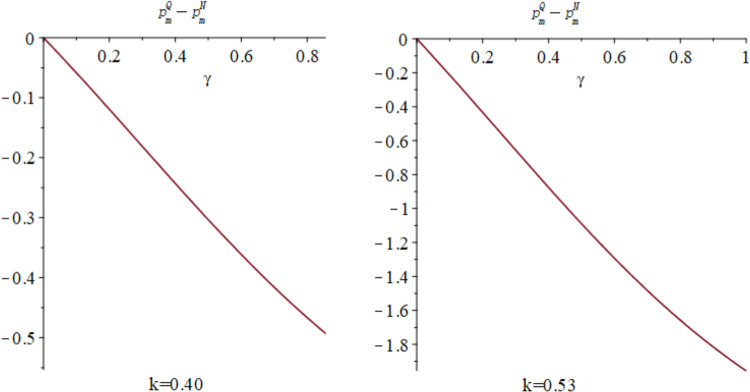
The impact of *γ* on $p_{m}^{Q*}-p_{m}^{N*}$ when *k* = 0.40 and *k* = 0.53.

**Fig 9:**
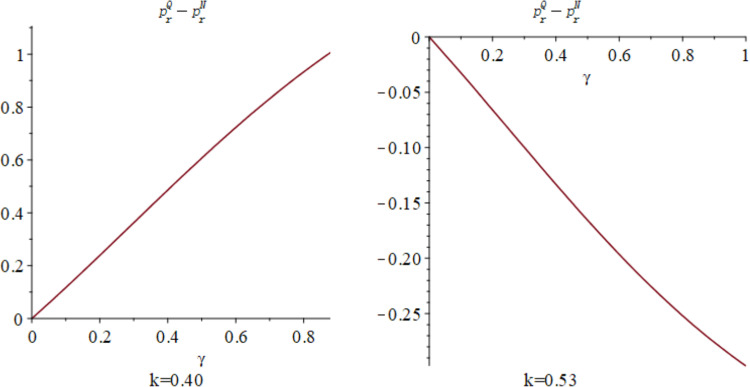
The impact of *γ* on $p_{r}^{Q*}-p_{r}^{N*}$ when *k* = 0.40 and *k* = 0.53.

Subsequently, the impact of quantum entanglement on manufacturers’ online sale prices and retailers’ offline retail prices under classical decentralized decision-making and quantum decision-making is explored, as illustrated in Fig 7. Fig 7 shows the influence of parameters *γ* and *k* on variables $p_{m}^{Q*}-p_{m}^{N*}$ and $p_{r}^{Q*}-p_{r}^{N*}$, and shows the change trend and relationship of $p_{r}^{Q*}-p_{r}^{N*}$ and $p_{r}^{Q*}-p_{r}^{N*}$ under different values of *γ* and *k*. It is evident from Fig 7 that entanglements between enterprises yield different effects on manufacturers’ online sale price decisions and retailers’ offline retail price decisions. Specifically, the online sales price under quantum decision-making consistently falls below that under decentralized decision-making, independent of consumers’ low-carbon preference and market competition. Conversely, the influence of quantum entanglement on retailers’ offline retail prices is contingent upon consumers’ low-carbon preference. Fig 8 and Fig 9 illustrates the sectional diagram of Fig 7 for *k* = 0.40 and *k* = 0.53. Fig 8 exhibits the impact of quantum entanglement on manufacturers’ online sale prices under quantum and decentralized decisions for consumers’ low-carbon preferences *k* = 0.40 and *k* = 0.53, respectively. Notably, regardless of the level of low-carbon preference, the discrepancy between them consistently remains less than 0 and increases with the degree of entanglement.

Fig 9 illustrates the impact of quantum entanglement on the offline retail prices of retailers under quantum and decentralized decision-making paradigms. In contrast to the pricing decisions of manufacturers, when *k* = 0.4, the difference between them exceeds 0, whereas when *k* = 0.53, the difference reduces to less than 0. With regard to the pricing decisions of manufacturers and retailers, it is evident that as entanglement intensifies, the price differential between online and offline retail prices increases under both quantum and decentralized decision-making frameworks.

**Fig 10:**
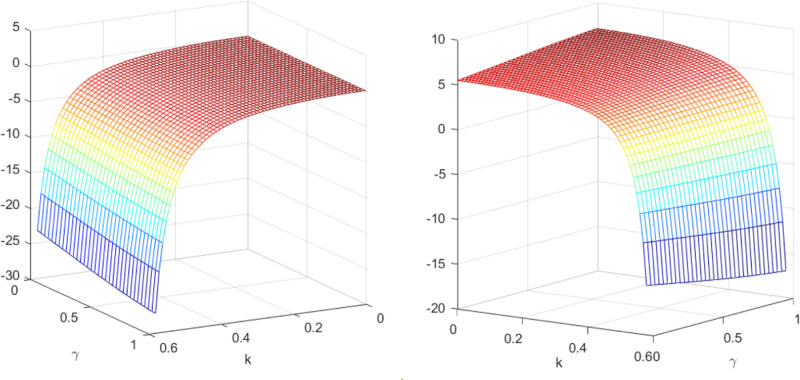
The impact of *γ* and *k* on $p_{m}^{Q*}-p_{m}^{C*}$ and $p_{r}^{Q*}-p_{r}^{C*}$.

**Fig 11:**
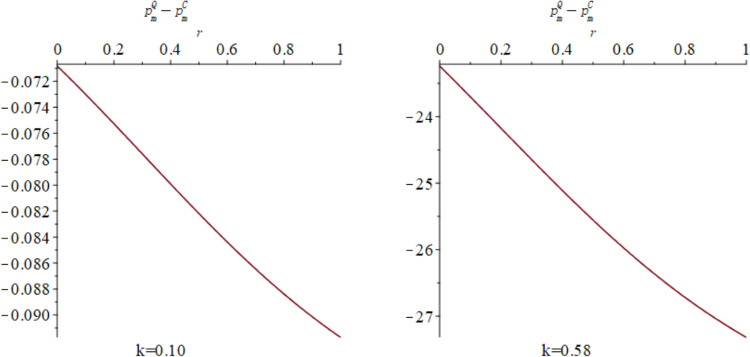
The impact of *γ* on $p_{m}^{Q*}-p_{m}^{C*}$ when *k* = 0.10 and *k* = 0.58.

**Fig 12:**
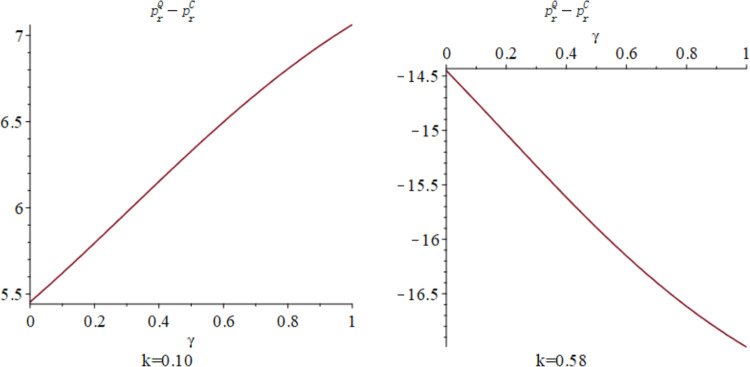
The impact of γ on $p_{r}^{Q*}-p_{r}^{C*}$ when *k* = 0.10 and *k* = 0.58.

Finally, Figs 10 respectively exhibit the influence of quantum entanglement on the online sale prices of manufacturers and the offline retail prices of retailers under centralized and quantum decision-making modalities. Fig 10 shows the influence of parameters *γ* and *k* on variables $p_{m}^{Q*}-p_{m}^{C*}$ and $p_{r}^{Q*}-p_{r}^{C*}$, and shows the change trend and relationship of $p_{m}^{Q*}-p_{m}^{C*}$ and $p_{r}^{Q*}-p_{r}^{C*}$ under different values of *γ* and *k*. For manufacturers, the optimal online sale price under quantum decision-making is discernibly lower than that under centralized decision-making. Fig 11 and Fig 12 illustrates the sectional diagram of Fig 10 for *k* = 0.10 and *k* = 0.58. Fig 11 As depicted in Fig 11, irrespective of the value of k, the differential is consistently less than 0. In contrast to the optimal pricing decision of manufacturers, the differential between the optimal offline retail price for retailers under quantum decision-making and centralized decision-making is contingent upon consumers’ proclivity for low-carbon products and the competitive dynamics of the market. As evidenced in Fig 12, on the basis of *β*=0.6, when $k<\sqrt{\frac{2}{3}-\frac{2\beta }{3}}$, the differential is positive; conversely, it is negative. Nevertheless, irrespective of consumers’ low-carbon preferences and market competition, the differential augments with increasing entanglement.

#### 4.3.3. Profit decision.

**Fig 13:**
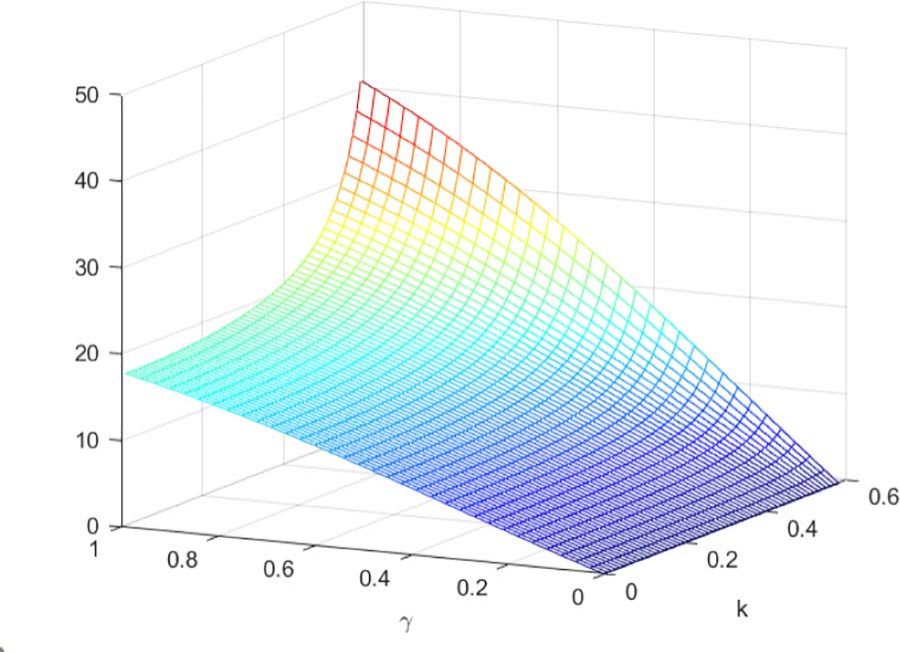
The impact of *γ* and *k* on $\pi_{m}^{Q*}-\pi_{m}^{N*}$.

**Fig 14:**
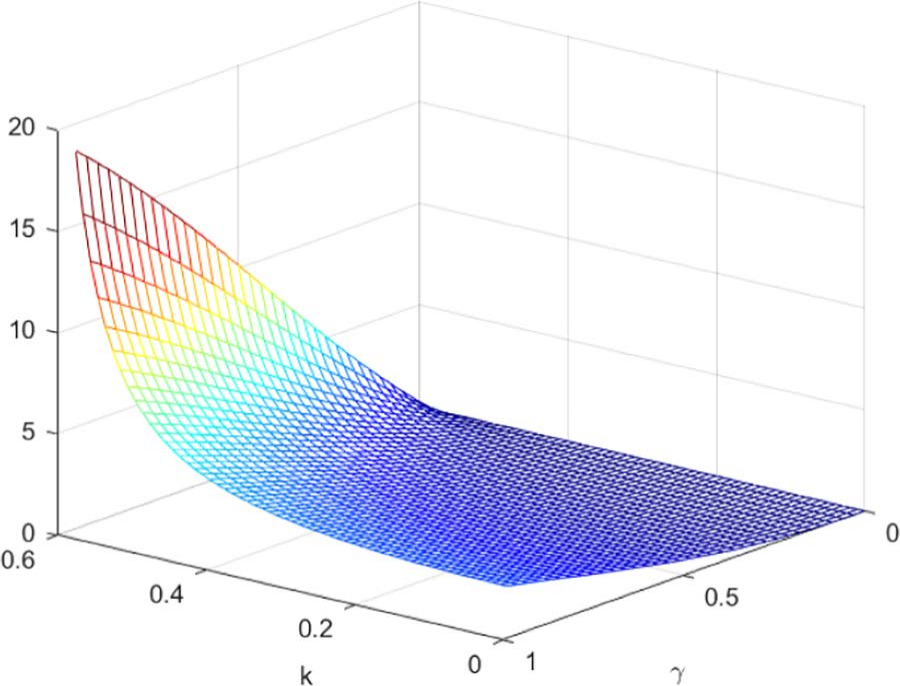
The impact of γ and *k* on $\pi_{r}^{Q*}-\pi_{r}^{N*}$.

Next, we make a comparative analysis of corporate profits. Fig 13 and Fig 14 shows the influence of parameters *γ* and *k* on variables $\pi_{m}^{Q*}-\pi_{m}^{N*}$ and $\pi_{r}^{Q*}-\pi_{r}^{N*}$, and shows the change trend and relationship of $\pi_{m}^{Q*}-\pi_{m}^{N*}$ and $\pi_{r}^{Q*}-\pi_{r}^{N*}$ under different values of *γ* and *k*. As depicted in Fig 13 and Fig 14, the optimal profit of manufacturers and retailers under the quantum strategy exceeds that of the decentralized decision. With the increase of entanglement, the disparity between the manufacturer’s optimal profit under the quantum strategy and the decentralized decision reduces, while the gap between gap retailer’s optimal profit under the quantum strategy and the decentralized decision increases. In contrast to the impact of entanglement on optimal price decisions, an increase in entanglement results in a reduction in the manufacturer’s profit and an increase in the retailer’s profit. Nevertheless, the total profit of both the manufacturer and the retailer in the quantum strategy experiences an augmentation with the increase in entanglement. This suggests that the degree of entanglement exerts a more pronounced effect on the retailer’s optimal profit, which diverges from its impact on optimal price decisions.

**Fig 15:**
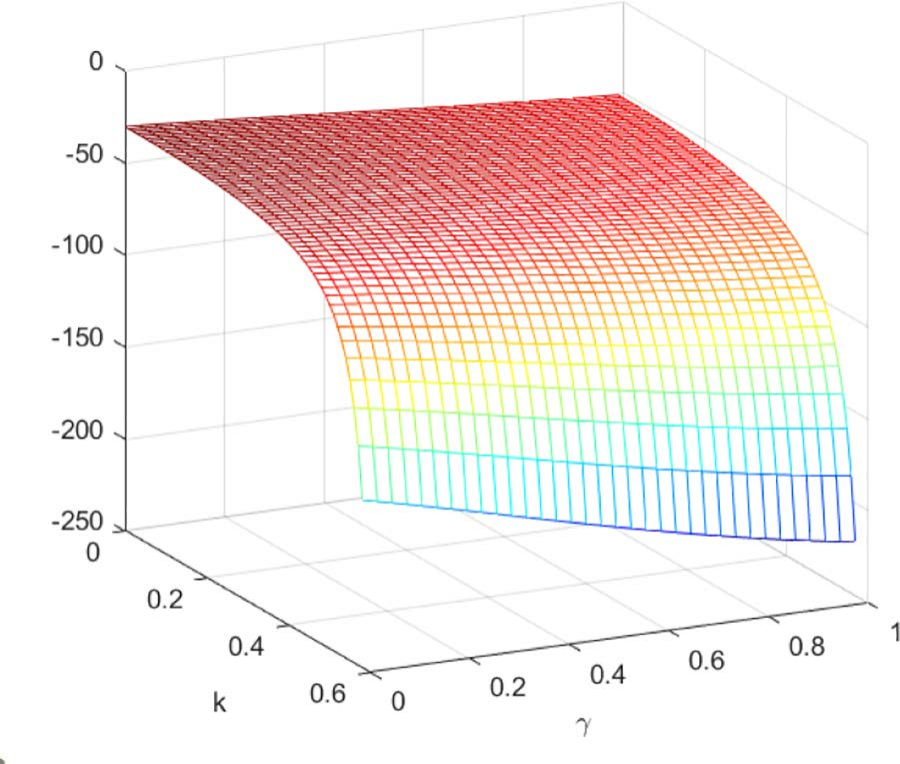
The impact of *γ* and *k* on $\pi_{m}^{Q*}+\pi_{r}^{Q*}-\pi^{C*}$.

**Fig 16:**
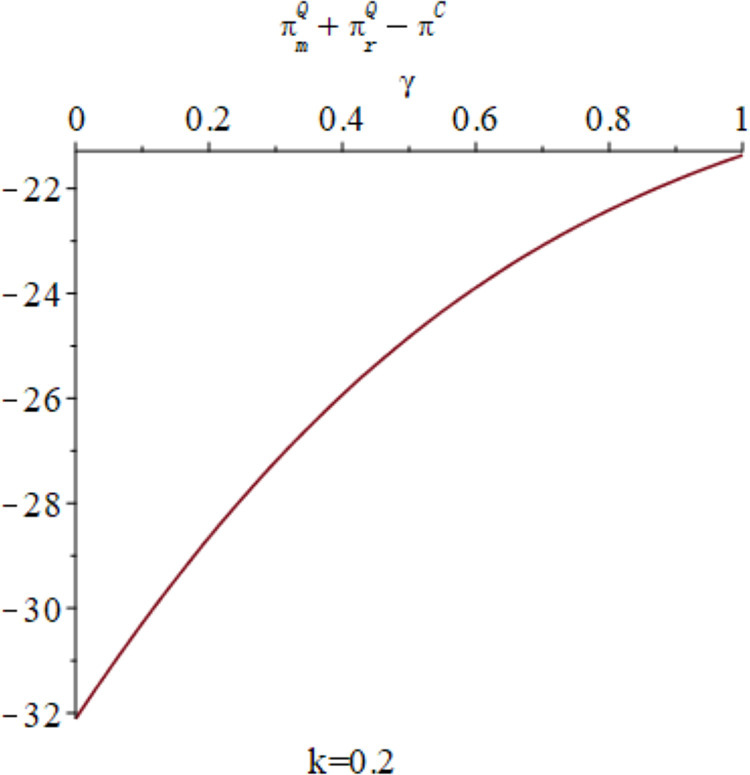
The impact of *γ* on $\pi_{m}^{Q*}+\pi_{r}^{Q*}-\pi^{C*}$ when *k* = 0.2.

In the context of centralized decision-making, it is observed that there is no quantum entanglement between the two enterprises, and the result of the quantum game aligns with that of the classical game. Fig 15 shows the relationship between the firm’s total profit under quantum strategy and centralized decision making. Fig 15 shows the influence of parameters *γ* and *k* on variables $\pi_{m}^{Q*}+\pi_{r}^{Q*}-\pi^{C*}$, and shows the change trend and relationship of $\pi_{m}^{Q*}+\pi_{r}^{Q*}-\pi^{C*}$ under different values of *γ* and *k*. As depicted in Fig 15, the aggregate profit realized by the manufacturer and retailer through the employment of quantum strategy is inferior to the total profit achieved under centralized decision. Nevertheless, as the degree of entanglement increases, the difference between the total profit derived from quantum decision-making and centralized decision gradually reduces, as illustrated in Fig 16. Fig 16 illustrates the sectional diagram of Fig 15 for *k* = 0.20. The impact of quantum entanglement on the manufacturer’s profit is negative, while it is positive for the retailer’s profit and the overall profit of the supply chain. Notably, as the degree of entanglement approaches infinity, the overall optimal profit derived from a quantum game surpasses that of classical games.

## 5. Conclusion

In consideration of consumers’ preferences towards low-carbon products and channel preferences, this study formulates a dual-channel supply chain game model wherein manufacturers establish online channels for the distribution of low-carbon goods. Three optimal low-carbon supply chain strategies are derived under centralized decision (*C*) and decentralized decision (Nash equilibrium (*N*) and quantum game equilibrium (*Q*)), taking into account the superposition and entanglement of quantum systems. It is evident that the quantization framework often yields superior pricing decisions and enhanced profits compared to classical games.

The analysis of the model and numerical examples yields the following conclusions: Firstly, upon comparison of the optimal low-carbon level of the manufacturer under the three strategies, it is concluded that in the decentralized decision mode, the Low-carbon level of the quantum game equilibrium (*Q*) surpasses that of the Nash equilibrium (*N*) irrespective of the entanglement level. Notably, as entanglement approaches infinity, the low-carbon level of the quantum game equilibrium exceeds that of the centralized decision. This phenomenon appears to be independent of consumers’ low-carbon preference and market competition. Evidently, entanglement in quantum games demonstrates a capacity to effectively enhance the low-carbon level of production by manufacturers. Secondly, from the vantage point of quantum games, it is identified that when entanglement is at 0, the optimal pricing of quantum games aligns with that of classical decentralized decisions. However, as entanglement varies, the pricing of quantum games also undergoes changes. Notably, quantum entanglement can markedly reduce manufacturers’ optimal wholesale and online sale prices. In addition, its impact on retailers’ optimal offline retail price is contingent upon consumers’ low-carbon preferences and market competition. It is evident that the optimal price of manufacturers and retailers exhibits differential sensitivity to entanglement. Finally, the optimal profit of the manufacturer and retailer under quantum game equilibrium (*Q*) surpasses that of the Nash equilibrium (*N*) of decentralized decision. Particularly, as entanglement tends to infinity, the total profit of the manufacturer and retailer under quantum game equilibrium outperforms the total profit under centralized decisions.

## 6. Managerial implications and future researches

### 6.1. Managerial implications

In today’s rapidly changing market environment, businesses need to cope with intense competition and changing consumer needs. Quantum game theory provides a new way to solve the uncertainties and conflicts in traditional game models, especially in the pricing and supply chain management of low-carbon products. By introducing properties such as quantum entanglement, quantum games can optimize pricing strategies and cooperation mechanisms, helping companies make more precise decisions in dynamic markets.

Specifically, (1) Because consumer demand for low carbon is constantly changing, and the degree of competition in different markets varies, retailers need to be flexible when setting prices to respond to these changes. Therefore, when making pricing strategies, managers should fully consider the degree of information exchange with competitive enterprises, so as to formulate more refined and dynamically adjusted pricing strategies. This can not only promote the sale of low-carbon products to meet the environmental needs of consumers, but also improve the economic efficiency and sustainable development level of the entire supply chain by optimizing the price system and resource allocation.

(2) The degree of entanglement between enterprises reflects the correlation between the earnings of both parties, and quantifying this correlation can better describe the market status. Before choosing a cooperation strategy, enterprises should use advanced market research tools, data mining techniques and business intelligence analysis methods to evaluate potential partners in the market and external market characteristics to clarify relevant attributes and their interrelationships (such as competitive landscape, key player information, product substitution in the end market, etc.). Especially for companies with influence and synergy potential in the market, companies should ensure that both parties are motivated by the same cooperation strategy based on observable and quantifiable performance indicators. In the process of contract design, we can set up appropriate incentive mechanism to stimulate the enthusiasm of cooperation between the two parties, so as to enhance the interaction and synergistic effect of each other.

(3) Retailers and manufacturers should optimize their pricing and cooperation strategies through quantum game models. Retailers need to adjust pricing flexibly according to consumers’ low-carbon preferences and changes in market competition, while strengthening cooperation with manufacturers to enhance the market penetration of low-carbon products by jointly developing incentive mechanisms and promotion strategies. Manufacturers should use the quantum game model to optimize wholesale prices and online sales prices, reduce cost pressure, and increase research and development investment in low-carbon products to enhance market competitiveness. The two should strengthen cooperation through entanglement contracts and incentive mechanisms to ensure supply chain coordination and profit maximization, promote the sale of low-carbon products, and improve the overall economic benefits.

### 6.2. Future researches

The study of co-operative relationship from the perspective of quantum strategy is a relatively new research field, and there is still a lot of room for expansion in future research. The subsequent research can be started from the following aspects.

Firstly, real market competition usually involves multiple enterprises, but this study only considers two enterprises with cooperative competition relationship. As the number of participating firms increases, the competitive landscape of the market may change, thus affecting the optimal strategy of firms in competitive and cooperative relationships. Therefore, the future research can be expanded to the “one-to-two” or “many-to-many” game structure on the basis of the current “one-to-one” to further enrich the research on the competition and cooperation relationship under different industries and market conditions.

Secondly, it may be interesting to explore the widely used stochastic update rule [50,51] in a low-carbon economy, and in particular how they affect corporate decisions on carbon reduction and resource allocation. By simulating different rules, evaluating their impact on supply chain efficiency and market response, and optimizing these rules to improve practical application effects, it provides new ideas for low-carbon economy policies and corporate strategies.

Finally, the concepts of quantum decoherence and quantum superposition deserve to be introduced into the decision-making analysis of enterprises in low-carbon supply chains. Quantum decoherence may affect the stability of decisions, while quantum superposition helps describe the flexibility of an enterprise under multiple choices. Considering these quantum effects may reveal new and interesting results for optimizing decision-making in a low-carbon economy.

## Supporting information

S1 Appendix Proofs of the main results. (DOCX)

## References

[1] Fan X, Chen K, Chen Y J. Is price commitment a better solution to control carbon emissions and promote technology investment? Manage Sci. 2023; 69(1): 325–41.

[2] Huang H, Yi M. Impacts and mechanisms of heterogeneous environmental regulations on carbon emissions: An empirical research based on DID method. Environmental Impact Assessment Review. 2023;99:107039. doi: 10.1016/j.eiar.2023.107039

[3] Xu C, Tang X, Song J, Wang C. Research on low-carbon dual channel supply chain considering product substitution under government carbon tax and low-carbon subsidy. PLoS One. 2023;18(6):e0287167. doi: 10.1371/journal.pone.0287167 37327248 PMC10275478

[4] Peng J, Li W, Li Y, et al. Innovative product design method for low-carbon footprint based on multi-layer carbon footprint information. J Clean Prod. 2019;228:729–45.

[5] Ma J. Carbon emissions and low-carbon innovation in firms. PLoS One. 2024;19(10):e0312759. doi: 10.1371/journal.pone.0312759 39446859 PMC11500900

[6] Dong Z, Zhang Z, Zhang F. Coupling coordination development of energy-economy-carbon emissions in China under the background of “double carbon”. PLoS One. 2022;17(12):e0277828. doi: 10.1371/journal.pone.0277828 36469512 PMC9721482

[7] Nie H, Zhou T, Lu H, Huang S. Evaluation of the efficiency of Chinese energy-saving household appliance subsidy policy: An economic benefit perspective. Energy Policy. 2021;149:112059. doi: 10.1016/j.enpol.2020.112059

[8] Liang P, Lv Y, Zhao Y. Incentive-compatible mechanism for manufacturing carbon emission supervision under carbon control policies in China. PLoS One. 2024;19(5):e0299086. doi: 10.1371/journal.pone.0299086 38739883 PMC11090604

[9] Gao F, Souza G C. Carbon offsetting with eco-conscious consumers. Manage Sci. 2022; 68(11):7879–97.

[10] Han X, Khouja M, Liu X. A dynamic model considering consumer green awareness and environmental subsidy. Int J Prod Econ. 2023;260:108840

[11] Lopes JM, Morales CC, Alvarado M, Melo VAZC, Paiva LB, Dias EM, et al. Optimization methods for large-scale vaccine supply chains: a rapid review. Ann Oper Res. 2022;316(1):699–721. doi: 10.1007/s10479-022-04720-5 35531563 PMC9059697

[12] Arruda EF, Das SS, Dias CM, Pastore DH. Modelling and optimal control of multi strain epidemics, with application to COVID-19. PLoS One. 2021;16(9):e0257512. doi: 10.1371/journal.pone.0257512 34529745 PMC8445490

[13] Meyer D A Quantum Strategies. Phys Rev Lett. 1999;82(5);1052–5

[14] Li M, Mizuno S. Dynamic pricing and inventory management of a dual-channel supply chain under different power structures. Eur J Oper Res. 2022; 303(1): 273–85.

[15] Tran M T, Rekik Y, Hadj-Hamou K. Optimal pricing for dual-channel retailing with stochastic attraction demand model. Int J Prod Econ. 2024;268:109127.

[16] Datta A, Sarkar B, Dey B K, et al. The impact of sales effort on a dual-channel dynamical system under a price-sensitive stochastic demand. J Retail Consum Serv. 2024;76:103561

[17] Heydari J, Govindan K, Basiri Z. Balancing price and green quality in presence of consumer environmental awareness: A green supply chain coordination approach. Int J Prod Res. 2021; 59(7): 1957–75.

[18] Li J, Luo X, Wang Q, Zhou W. Supply chain coordination through capacity reservation contract and quantity flexibility contract. Omega. 2021;99:102195. doi: 10.1016/j.omega.2020.102195

[19] Zhao L, Guo W, Fang S. C., An Q. Enhancing supply chain coordination through transparency initiatives to mitigate product returns. J Retail Consum Serv. 2024;78;103756.

[20] Song J M, Zhao Y. Supply chain coordination for e-commerce: Risk penalty vs. flat rate. Manuf Serv Oper Manag. 2022; 24(2): 1110–27.

[21] Zhang Y, Xu Q. Agency contracts with incentive mechanisms considering supplier risk aversion in a dynamic platform supply chain. Ann Oper Res. 2024; 1–31.

[22] Feng X, Zhao Y, Yan R. Does carbon emission trading policy has emission reduction effect?-An empirical study based on quasi-natural experiment method. J Environ Manage. 2024;351:119791. doi: 10.1016/j.jenvman.2023.119791 38128208

[23] Chen J, Sun C, Wang Y, Liu J, Zhou P. Carbon emission reduction policy with privatization in an oligopoly model. Environ Sci Pollut Res Int. 2023;30(15):45209–30. doi: 10.1007/s11356-022-24256-2 36705827

[24] Cai J, Jiang F. Decision models of pricing and carbon emission reduction for low-carbon supply chain under cap-and-trade regulation. Int J Prod Econ. 2023;264:108964.

[25] Ghosh S K, Seikh M R, Chakrabortty M. Analyzing a stochastic dual-channel supply chain under consumers’ low-carbon preferences and cap-and-trade regulation. Comput Ind Eng. 2020;149: 106765.

[26] Zhu J, Feng T, Lu Y, Jiang W. Using blockchain or not? A focal firm’s blockchain strategy in the context of carbon emission reduction technology innovation. Bus Strat Env. 2024;33(4):3505–31. doi: 10.1002/bse.3664

[27] Ding J, Chen W, Wang W. Production and carbon emission reduction decisions for remanufacturing firms under carbon tax and take-back legislation. Comput Ind Eng. 2020;143;106419

[28] Yu W, Wang Y, Feng W, Bao L, Han R. Low-carbon strategy analysis with two competing supply chain considering carbon taxation. Comput Ind Eng. 2022;169:108203

[29] Wang Y, Xu X, Zhu Q. Carbon emission reduction decisions of supply chain members under cap-and-trade regulations: A differential game analysis. Comput Ind Eng. 2021;162:107711.

[30] Li W, Wang P, Cheng W, Nie K. Transnational remanufacturing decisions under carbon taxes and tariffs. Eur J Oper Res. 2023; 312(1):150–163.

[31] Liu Z, Lang L, Hu B. Emission reduction decision of agricultural supply chain considering carbon tax and investment cooperation. J Clean Prod. 2021; 294:126305.

[32] Zhang C, Xing P, Wang J. Quality effort decision in service supply chain with quality preference based on quantum game. Int J Mod Phys C. 2015; 26(07):1550073.

[33] Wu A, Li H, Du D. Quantum game analysis of green technology R&D cooperation between competing manufacturers under government subsidies. Technol Anal Strateg. 2022; 34:266.

[34] Babu S, Mohan U, Arthanari T. Modeling Coopetition as a Quantum Game. Int Game Theory Rev. 2020;22(02):2040001. doi: 10.1142/s0219198920400010

[35] Fadaki M, Abbasi B, Chhetri P. Quantum game approach for capacity allocation decisions under strategic reasoning. Comput Manag Sci. 2022; 1–22.

[36] He W, Zhang Y, Li S, et al. Reducing betrayal behavior in green building construction: A quantum game approach. J Clean Prod. 2024;463:142760.

[37] Peng B, Xu N, Luo R, Elahi E, Wan A. Promoting green investment behavior in “belt and road” energy projects: A quantum game approach. Technological Forecasting Social Change. 2024;204:123416. doi: 10.1016/j.techfore.2024.123416

[38] Zhang L, Chen F. Quantum Game‐Based Study on the Incentive Mechanism for the Cooperative Distribution of E‐Commerce Logistics Alliance. Discrete Dynamics Nature Society. 2024;2024(1). doi: 10.1155/2024/2590861

[39] Piotrowski E W, Sładkowski J. An invitation to quantum game theory. Int J Theor Phys. 2023(a); 42:1089-99.

[40] Du J, Li H, Xu X, Shi M, Wu J, Zhou X, et al. Experimental realization of quantum games on a quantum computer. Phys Rev Lett. 2002;88(13):137902. doi: 10.1103/PhysRevLett.88.137902 11955126

[41] Li Y, Zhao Y, Fu J, Xu L. Reducing food loss and waste in a two-echelon food supply chain: A quantum game approach. J Clean Prod. 2021(b); 285:125261.

[42] Akter M, Alam M, Kamrujjaman Md. An in-silico game theoretic approach for health intervention efficacy assessment. Healthcare Analytics. 2024;5:100318. doi: 10.1016/j.health.2024.100318

[43] Kulsum U, Alam M, Kamrujjaman Md. Modeling and investigating the dilemma of early and delayed vaccination driven by the dynamics of imitation and aspiration. Chaos, Solitons & Fractals. 2024;178:114364. doi: 10.1016/j.chaos.2023.114364

[44] Yang M, Zhang T, Zhang Y. Optimal pricing and green decisions in a dual-channel supply chain with cap-and-trade regulation. Environ Sci Pollut Res Int. 2022;29(19):28208–25. doi: 10.1007/s11356-021-18097-8 34993817

[45] Piotrowski E W, Sładkowski J. Trading by quantum rules: Quantum anthropic principle. Int J Theor Phys. 2003(b); 42:1101–6.

[46] Ma D, Hu J, Yao F. Big data empowering low-carbon smart tourism study on low-carbon tourism O2O supply chain considering consumer behaviors and corporate altruistic preference. Comput Ind Eng. 2021; 153: 107061.

[47] Shi P, Wang J, Yan B. Decision-making of the Multi-channel Low-carbon Tourism Supply Chain under a Competitive Environment with Fairness Concern. J Syst Sci Syst Eng. 2024;1–32.

[48] Wang M, Liu Y, Shi W. Research on technology remote synergic sharing strategy of low carbon under the ETS in China. Syst-Eng-Theory Pract. 2019;39:1419–34.

[49] Alam M, Tanimoto J. A Game-Theoretic Modeling Approach to Comprehend the Advantage of Dynamic Health Interventions in Limiting the Transmission of Multi-Strain Epidemics. J Appl Mathematics Phys. 2022;10(12):3700–48. doi: 10.4236/jamp.2022.1012248

[50] Fu F, Rosenbloom DI, Wang L, Nowak MA. Imitation dynamics of vaccination behaviour on social networks. Proc Biol Sci. 2011;278(1702):42–9. doi: 10.1098/rspb.2010.1107 20667876 PMC2992723

[51] Alam M, Kuga K, Tanimoto J. Three-strategy and four-strategy model of vaccination game introducing an intermediate protecting measure. Appl Math Comput. 2019; 346: 408–22.

