# Supplementary material for: Optimizing low-carbon strategies in dual-channel supply chains: A quantum game perspective
Source: PLoS One. 2025 Jun 2;20(6):e0323564. doi: 10.1371/journal.pone.0323564 (PMC12129178; doi:10.1371/journal.pone.0323564)
Supplement: S1 Appendix — (DOCX) [file pone.0323564.s001.docx]

**Proof of Proposition 1.**

Take the first derivatives of $e^{C*}$,$p_{m}^{C*}$,$p_{r}^{C*}$, and $\pi^{C*}$ with respect to *β* and *k*, respectively. We can get $\frac{\partial e^{C*}}{\partial\beta}=\frac{k\left(2\alpha-ck^{2}\right)}{2\left(k^{2}+\beta-1\right)^{2}}$, $\frac{\partial e^{C*}}{\partial k}=\frac{(2\alpha+c\beta-c)\left(k^{2}-\beta+1\right)}{2\left(k^{2}+\beta-1\right)^{2}}$,$\frac{\partial p_{m}^{C*}}{\partial\beta}=\frac{4\left(2\alpha-ck^{2}\right)}{\left(4k^{2}+4\beta-4\right)^{2}}$, $\frac{\partial p_{m}^{C*}}{\partial k}=\frac{(\beta-1)c+2\alpha}{2\left(k^{2}+\beta-1\right)^{2}}k$, $\frac{\partial p_{r}^{C*}}{\partial\beta}=\frac{2\alpha-ck^{2}}{4\left(k^{2}+\beta-1\right)^{2}}$, $\frac{\partial p_{r}^{C*}}{\partial k}=\frac{(\beta-1)c+2\alpha}{2\left(k^{2}+\beta-1\right)^{2}}k\text{，}$ $\frac{\partial\pi^{C*}}{\partial\beta}=\frac{\left(ck^{2}-2\alpha\right)^{2}}{8\left(k^{2}+\beta-1\right)^{2}}$ and $\frac{\partial\pi^{C*}}{\partial k}=\frac{((\beta-1)c+2\alpha)^{2}}{4\left(k^{2}+\beta-1\right)^{2}}k$.

According to $p_{r}^{C*}>0$, we get $2\alpha-ck^{2}>0$, so $\frac{\partial e^{C*}}{\partial\beta}>0$, $\frac{\partial p_{m}^{C*}}{\partial\beta}>0$,$\frac{\partial p_{r}^{C*}}{\partial\beta}>0$; Obviously, $\frac{\partial\pi^{C*}}{\partial\beta}>0$, $\frac{\partial p_{m}^{C*}}{\partial k}>0$, $\frac{\partial p_{r}^{C*}}{\partial k}>0$, $\frac{\partial\pi^{C*}}{\partial k}>0$; According to $e^{C*}>0$, we get $2\alpha+c\beta-c>0$， and because of $1+k^{2}>\beta$, $\frac{\partial e^{C*}}{\partial k}>0$. $p_{m}^{C*}-p_{r}^{C*}=-\frac{2(k^{2}+\beta-1)c}{4\left(1-k^{2}-\beta\right)}=\frac{c}{2}>0$.

**Proof of Proposition 2.**

Take the first derivatives of $w^{N*}$, $e^{N*}$, $p_{m}^{N*}$, $p_{r}^{N*}$ and $\pi_{m}^{N*}$, $\pi_{r}^{N*}$ with respect to *β*, respectively. We can get $\frac{\partial w^{N*}}{\partial\beta}=\frac{4(4+k^{2})\alpha-(12-k^{2})k^{2}c}{2\left((\beta+3)k^{2}+4\beta-4\right)^{2}}$. let $\text{s}_{1}=\frac{(12-k^{2})}{(4+k^{2})}k^{2}$, and $\frac{\partial\text{s}_{1}}{\partial k^{2}}=\frac{48-8k^{2}-k^{4}}{(4+k^{2})} >0$，because of $\text{0<}k^{2}<\frac{4(1-\beta)}{3+\beta}$, $(\text{s}_{1})_{\max}=s_{1}(\frac{4(1-\beta)}{3+\beta})=\frac{4(1-\beta)(2+\beta)}{3+\beta}$，according to $e^{N*}>0$ and $4\alpha>(\text{s}_{1})_{\max}c$，we get$\frac{\partial w^{N*}}{\partial\beta}=(4+k^{2})\frac{4\alpha-\frac{(12-k^{2})}{(4+k^{2})}k^{2}c}{2\left((\beta+3)k^{2}+4\beta-4\right)^{2}}>\frac{(4+k^{2})\left(4\alpha-(s_{1})_{\max}c\right)}{2\left((\beta+3)k^{2}+4\beta-4\right)^{2}}>0$.

The same way, $\frac{\partial p_{m}^{N*}}{\partial\beta}\frac{4(k^{2}+4)\alpha+(k^{2}-12)k^{2}c}{2\left((\beta+3)k^{2}+4\beta-4\right)^{2}}>0$.$\frac{\partial e^{N*}}{\partial\beta}=k\frac{16\alpha-\left[(\beta+1)(\beta+5)k^{2}+4(\beta-1)^{2}\right]c}{\left(\beta k^{2}+3k^{2}+4\beta-4\right)^{2}}$，because of $\text{0<}k^{2}<\frac{4(1-\beta)}{3+\beta}$ and$(\beta+1)(\beta+5)k^{2}+4(\beta-1)^{2}<(\beta+1)(\beta+5)\frac{4(1-\beta)}{3+\beta}+4(1-\beta)^{2}=4(1-\beta)\frac{4(\beta+2)}{3+\beta}$, we according to$\alpha>\frac{(1-\beta)(\beta+2)c}{(3+\beta)}$ get $\frac{\partial e^{N*}}{\partial\beta}>0$.

$\frac{\partial p_{r}^{N*}}{\partial\beta}=\frac{4(3k^{2}+4)\alpha+\left(3k^{4}+2\left(\beta^{2}+6\beta-13\right)k^{2}+8(1-\beta)^{2}\right)c}{2\left((\beta+3)k^{2}+4\beta-4\right)^{2}}$， let $s_{2}=\frac{-3k^{4}-2\left(\beta^{2}+6\beta-13\right)k^{2}-8(1-\beta)^{2}}{(3k^{2}+4)}$, we get $\frac{\partial s_{2}}{\partial k^{2}}=\frac{16\beta^{2}-9k^{4}-96\beta-24k^{2}+128}{(3k^{2}+4)^{2}}>0$. $(s_{2})_{\max}=s_{2}(\frac{4(1-\beta)}{3+\beta})=\frac{4(2+\beta)(1-\beta)}{3+\beta}$， so $\frac{\partial p_{r}^{N*}}{\partial\beta}>0$.

$\frac{\partial\pi_{\text{m}}^{N*}}{\partial\beta}=\frac{\left(\begin{matrix} 16\alpha^{2}-2\left((\beta^{2}+6\beta+5)k^{2}+4(\beta^{2}-2\beta+1)\right)\alpha c+ \\ \left(k^{4}+(-2\beta^{3}-8\beta^{2}+6\beta+4)k^{2}-8\beta^{3}+16\beta^{2}-8\beta\right)c^{2} \end{matrix}\right)}{2\left(\beta k^{2}+3k^{2}+4\beta-4\right)^{2}}$. Set$s_{3}=16\alpha^{2}-2\left((\beta^{2}+6\beta+5)k^{2}+4(\beta^{2}-2\beta+1)\right)\alpha c+\left(k^{4}+(-2\beta^{3}-8\beta^{2}+6\beta+4)k^{2}-8\beta^{3}+16\beta^{2}-8\beta\right)c^{2}$, we get $\frac{\partial s_{3}}{\partial k}=-4(\beta^{2}+6\beta+5)\left(\alpha-\frac{\left(k^{2}-\beta^{3}-4\beta^{2}+3\beta+2\right)}{\beta^{2}+6\beta+5}c\right)kc$， because of$\alpha-\frac{\left(k^{2}-\beta^{3}-4\beta^{2}+3\beta+2\right)}{\beta^{2}+6\beta+5}c>\alpha-\frac{(\beta+2)(1-\beta)}{3+\beta}c>0$ and $\frac{\partial s_{3}}{\partial k}<0$， so $s_{3}>(s_{3})_{\max}\text{=}s_{3}(0)=16\alpha^{2}-8[\alpha+\beta c](\beta^{2}-2\beta+1)c>0$. The same way, according to $\frac{\partial\pi_{r}^{N*}}{\partial\beta}=\frac{\left(2(1-\beta)\alpha-\left(\beta k^{2}+k^{2}+2\beta^{2}-2\beta\right)c\right)\left(4k^{2}\alpha+\left(k^{4}+(\beta^{2}+6\beta-7)k^{2}+4(\beta^{2}-2\beta+1)\right)c\right)}{\left(4(1-\beta)-(\beta+3)k^{2}\right)^{3}}$, We can get .

Take the first derivatives of $w^{N*}$, $e^{N*}$, $p_{m}^{N*}$, $p_{r}^{N*}$ and $\pi_{m}^{N*}$, $\pi_{r}^{N*}$ with respect to *k*, respectively. We can get $\frac{\partial w^{N*}}{\partial k}=4\frac{(3+\beta)\alpha-(2-\beta^{2}-\beta)c}{\left(\beta k^{2}+3k^{2}+4\beta-4\right)^{2}}k>0$, $\frac{\partial e^{N*}}{\partial k}=\frac{\left((\beta+3)\alpha-(2-\beta^{2}-\beta)c\right)\left(4(1-\beta)+\beta k^{2}+3k^{2}\right)}{\left(\beta k^{2}+3k^{2}+4\beta-4\right)^{2}}>0$, $\frac{\partial p_{m}^{N*}}{\partial k}=\frac{4\left((\beta+3)\alpha-(2-\beta^{2}-\beta)c\right)k}{\left(\beta k^{2}+3k^{2}+4\beta-4\right)^{2}}>0$, $\frac{\partial p_{r}^{N*}}{\partial k}=\frac{2\left((\beta+3)\alpha-(2-\beta^{2}-\beta)c\right)(3-\beta)k}{\left(\beta k^{2}+3k^{2}+4\beta-4\right)^{2}}>0$ , $\frac{\partial\pi_{\text{m}}^{N*}}{\partial k}=\frac{\left(\beta^{2}c+(c+\alpha)\beta-2c+3\alpha\right)^{2}k}{\left(\beta k^{2}+3k^{2}+4\beta-4\right)^{2}}>0$, , $p_{m}^{N*}-p_{r}^{N*}=-\frac{(1-\beta)\alpha-(\beta^{2}-3\beta-k^{2}+2)c}{4(1-\beta)-(3+\beta)k^{2}}<0$ and $\frac{\partial\pi_{r}^{N*}}{\partial k}=-\frac{2\left((\beta+3)\alpha-(2-\beta^{2}-\beta)c\right)\left(2(1-\beta)\alpha-(2\beta^{2}-2\beta+k^{2}+\beta k^{2})c+\right)(1-\beta)k}{\left(\beta k^{2}+3k^{2}+4\beta-4\right)^{3}}>0$.

**Proof of Corollary 1.**

Take the first derivatives of $w^{Q*}$, $e^{Q*}$, $p_{m}^{Q*}$, $p_{r}^{Q*}$ and $\pi_{m}^{Q*}$, $\pi_{r}^{Q*}$ with respect to *β*, respectively.

Let $S=\left(\begin{matrix} \left(4\beta^{4}+\left(8k^{2}-8\right)\beta^{3}+5\left(k^{4}+4\right)\beta^{2}+\left(6k^{4}+16k^{2}-32\right)\beta+9\left(k^{2}-\frac{4}{3}\right)^{2}\right)\cosh^{2}\gamma- \\ 4\beta\left(k^{2}+\beta-1\right)\left(\beta k^{2}+3k^{2}+4\beta-4\right)\sinh\gamma\cosh\gamma-4\beta^{2}\left(k^{2}+\beta-1\right)^{2} \end{matrix}\right)$, we can get, $\frac{\partial e^{Q\text{*}}}{\partial r}=\frac{\left(2(1-\beta)\alpha-(2\beta^{2}+\beta k^{2}-2\beta+k^{2})c\right)k\beta(1-\beta)}{S}$, $\frac{\partial w^{Q\text{*}}}{\partial r}=\frac{-\left(\left(2(1-\beta)\alpha-(2\beta^{2}+\beta k^{2}-2\beta+k^{2})c\right)k^{2}\beta\right)}{2S}$, $\frac{\partial p_{m}}{\partial r}=\frac{\left(\left(2\beta^{2}c+\left(ck^{2}+2\alpha-2c\right)\beta+ck^{2}-2\alpha\right)k^{2}\beta\right)}{2S}$ and $\frac{\partial p_{r}}{\partial r}=\frac{\left(3\left(2\beta^{2}c+\left(ck^{2}+2\alpha-2c\right)\beta+ck^{2}-2\alpha\right)\beta\left(k^{2}+\frac{2\beta}{3}-\frac{2}{3}\right)\right)}{2S}$.

**Proof of Proposition 4.**

$\lim_{r\to\infty} e^{Q*}-e^{N*}=\frac{\beta\left(2\beta^{2}c+\left(ck^{2}+2\alpha-2c\right)\beta+ck^{2}-2\alpha\right)k(\beta-1)}{\left(\beta k^{2}+2\beta^{2}-3k^{2}-6\beta+4\right)\left(\beta k^{2}+3k^{2}+4\beta-4\right)}$,

$\lim_{r\to\infty} e^{Q*}-e^{C*}=\frac{\left(2\beta^{2}c+\left(ck^{2}+2\alpha-2c\right)\beta+ck^{2}-2\alpha\right)(\beta-1)k}{2\left(2\beta^{2}+\left(k^{2}-6\right)\beta-3k^{2}+4\right)\left(k^{2}+\beta-1\right)}$,

$e^{C*}-e^{Q*}=\frac{\left(2\beta^{2}c+\left(ck^{2}+2\alpha-2c\right)\beta+ck^{2}-2\alpha\right)\cosh(r)(\beta-1)k}{2\left(\left(\beta k^{2}+3k^{2}+4\beta-4\right)\cosh r-2\beta\left(k^{2}+\beta-1\right)\sinh r\right)\left(k^{2}+\beta-1\right)}$,

**Proof of Proposition 5.**

When $\gamma\to\infty$, we can get,

$\lim_{r\to\infty} w^{Q*}-w^{N*}=-\frac{\beta\left(2\beta^{2}c+\left(ck^{2}+2\alpha-2c\right)\beta+ck^{2}-2\alpha\right)k^{2}}{2\left(\beta k^{2}+2\beta^{2}-3k^{2}-6\beta+4\right)\left(\beta k^{2}+3k^{2}+4\beta-4\right)}$,

$\lim_{r\to\infty} p_{m}^{Q*}-p_{m}^{N*}\text{=}-\frac{\beta\left(2\beta^{2}c+\left(ck^{2}+2\alpha-2c\right)\beta+ck^{2}-2\alpha\right)k^{2}}{2\left(\beta k^{2}+2\beta^{2}-3k^{2}-6\beta+4\right)\left(\beta k^{2}+3k^{2}+4\beta-4\right)}$,

$\lim_{r\to\infty} p_{m}^{Q*}-p_{m}^{C*}=-\frac{\left(2\beta^{2}c+\left(ck^{2}+2\alpha-2c\right)\beta+ck^{2}-2\alpha\right)k^{2}}{4\left(2\beta^{2}+\left(k^{2}-6\right)\beta-3k^{2}+4\right)\left(k^{2}+\beta-1\right)}$,

$\lim_{r\to\infty} p_{r}^{Q*}-p_{r}^{N*}=-\frac{3\left(k^{2}+\frac{2\beta}{3}-\frac{2}{3}\right)\beta\left(2\beta^{2}c+\left(ck^{2}+2\alpha-2c\right)\beta+ck^{2}-2\alpha\right)}{2\left(\beta k^{2}+2\beta^{2}-3k^{2}-6\beta+4\right)\left(\beta k^{2}+3k^{2}+4\beta-4\right)}$,

$\lim_{r\to\infty} p_{r}^{Q*}-p_{r}^{C*}=-\frac{3\left(k^{2}+\frac{2\beta}{3}-\frac{2}{3}\right)\left(2\beta^{2}c+\left(ck^{2}+2\alpha-2c\right)\beta+ck^{2}-2\alpha\right)}{4\left(2\beta^{2}+\left(k^{2}-6\right)\beta-3k^{2}+4\right)\left(k^{2}+\beta-1\right)}$.

According to the above results, we examine decentralized decision Nash equilibrium (*N*) and quantum game equilibrium (*Q*),

$w^{Q*}-w^{N*}=\frac{\beta k^{2}\left(2\beta^{2}c+\left(ck^{2}+2\alpha-2c\right)\beta+ck^{2}-2\alpha\right)\sinh r}{2\left(\left(k^{2}\beta+3k^{2}+4\beta-4\right)\cosh r-2\beta\left(k^{2}+\beta-1\right)\sinh r\right)\left(k^{2}\beta+3k^{2}+4\beta-4\right)}$,

$p_{m}^{Q*}-p_{m}^{N*}=\frac{\left(2\beta^{2}c+\left(ck^{2}+2\alpha-2c\right)\beta+ck^{2}-2\alpha\right)\beta k^{2}\sinh\gamma}{2\left(\left(\beta k^{2}+3k^{2}+4\beta-4\right)\cosh\gamma-2\beta\left(k^{2}+\beta-1\right)\sinh\gamma\right)\left(\beta k^{2}+3k^{2}+4\beta-4\right)}$,

$p_{r}^{Q*}-p_{r}^{N*}=\frac{3\left(2\beta^{2}c+\left(ck^{2}+2\alpha-2c\right)\beta+ck^{2}-2\alpha\right)\beta\left(k^{2}+\frac{2\beta}{3}-\frac{2}{3}\right)\sinh\gamma}{2\left(\left(\beta k^{2}+3k^{2}+4\beta-4\right)\cosh\gamma-2\beta\left(k^{2}+\beta-1\right)\sinh\gamma\right)\left(\beta k^{2}+3k^{2}+4\beta-4\right)}$.

we examine decentralized decision Nash equilibrium (*N*) and centralized decision (*C*),

$p_{m}^{Q*}-p_{m}^{C*}=\frac{\left(2\beta^{2}c+\left(ck^{2}+2\alpha-2c\right)\beta+ck^{2}-2\alpha\right)k^{2}\cosh\gamma}{4\left(\left(\beta k^{2}+3k^{2}+4\beta-4\right)\cosh\gamma-2\beta\left(k^{2}+\beta-1\right)\sinh\gamma\right)\left(k^{2}+\beta-1\right)}$,

$p_{r}^{Q*}-p_{r}^{C*}=\frac{3\left(2\beta^{2}c+\left(ck^{2}+2\alpha-2c\right)\beta+ck^{2}-2\alpha\right)\left(k^{2}+\frac{2\beta}{3}-\frac{2}{3}\right)\cosh\gamma}{4\left(\left(\beta k^{2}+3k^{2}+4\beta-4\right)\cosh\gamma-2\beta\left(k^{2}+\beta-1\right)\sinh\gamma\right)\left(k^{2}+\beta-1\right)}$.

we examine decentralized decision quantum game equilibrium (*Q*) and centralized decision (*C*),

$p_{m}^{C*}-p_{m}^{N*}=-\frac{k^{2}\left(2\beta^{2}c+\left(ck^{2}+2\alpha-2c\right)\beta+ck^{2}-2\alpha\right)}{4\left(\left(k^{2}+4\right)\beta+3k^{2}-4\right)\left(k^{2}+\beta-1\right)}$,

$p_{r}^{C*}-p_{r}^{N*}=-\frac{3\left(2\beta^{2}c+\left(ck^{2}+2\alpha-2c\right)\beta+ck^{2}-2\alpha\right)\left(k^{2}+\frac{2\beta}{3}-\frac{2}{3}\right)}{4\left(\left(k^{2}+4\right)\beta+3k^{2}-4\right)\left(k^{2}+\beta-1\right)}$.

**Proof of Proposition 6.**

When $\lambda\to\infty$, we can set $S_{n}=2\beta^{2}c+\left(ck^{2}+2\alpha-2c\right)\beta+ck^{2}-2\alpha$, so

$\lim_{r\to\infty} \pi_{m}^{Q*}-\pi_{m}^{N*}=\frac{\beta\left(S_{n}\right)^{2}}{4\left(\beta k^{2}+2\beta^{2}-3k^{2}-6\beta+4\right)\left(\beta k^{2}+3k^{2}+4\beta-4\right)}$,

$\lim_{r\to\infty} \pi_{r}^{Q*}-\pi_{r}^{N*}=-\frac{\beta\left(4\beta^{3}+\left(k^{4}+12k^{2}-8\right)\beta^{2}+\left(6k^{4}-16k^{2}+4\right)\beta-3k^{4}+4k^{2}\right)\left(S_{n}\right)^{2}}{4\left(\beta k^{2}+2\beta^{2}-3k^{2}-6\beta+4\right)^{2}\left(\beta k^{2}+3k^{2}+4\beta-4\right)^{2}}$,

$\lim_{r\to\infty} (\pi_{m}^{Q*}+\pi_{r}^{Q*})-\pi^{C*}=\frac{\left(\left(k^{2}+2\right)\beta+k^{2}-2\right)\left(S_{n}\right)^{2}}{8\left(k^{2}+\beta-1\right)\left(2\beta^{2}+\left(k^{2}-6\right)\beta-3k^{2}+4\right)^{2}}$.

According to the above results, we examine decentralized decision Nash equilibrium (*N*) and quantum game equilibrium (*Q*),

$$\pi_{m}^{Q*}-\pi_{m}^{N*}=\frac{\left(S_{n}\right)^{2}\beta\sinh\gamma}{4\left(\beta k^{2}+3k^{2}+4\beta-4\right)\left(\left(\beta k^{2}+3k^{2}+4\beta-4\right)\cosh\gamma-2\beta\left(k^{2}+\beta-1\right)\sinh\gamma\right)}$$

$$\pi_{r}^{Q*}-\pi_{r}^{N*}=\frac{\left(k^{2}(\beta-1)\left(\beta k^{2}+4\beta+3k^{2}-4\right)\cosh\gamma+4\beta(k^{2}+\beta-1)^{2}\sinh\gamma\right)\left(S_{n}\right)^{2}\beta\sinh\gamma}{4(\beta k^{2}+3k^{2}+4\beta-4)^{2}S}$$

we examine decentralized decision quantum game equilibrium (*Q*) and centralized decision (*C*), $\pi_{m}^{Q*}+\pi_{r}^{Q*}-\pi^{C*}=-\frac{\cosh(\gamma)^{2}\left(\beta k^{2}+k^{2}+2\beta-2\right)\left(S_{n}\right)^{2}}{8(k^{2}+\beta-1)S}$
